# Export of malaria proteins requires co-translational processing of the PEXEL motif independent of phosphatidylinositol-3-phosphate binding

**DOI:** 10.1038/ncomms10470

**Published:** 2016-02-01

**Authors:** Justin A. Boddey, Matthew T. O'Neill, Sash Lopaticki, Teresa G. Carvalho, Anthony N. Hodder, Thomas Nebl, Stephan Wawra, Pieter van West, Zeinab Ebrahimzadeh, Dave Richard, Sven Flemming, Tobias Spielmann, Jude Przyborski, Jeff J. Babon, Alan F. Cowman

**Affiliations:** 1 The Walter and Eliza Hall Institute of Medical Research, 1G Royal Parade, Parkville, Victoria 3052, Australia; 2 Department of Medical Biology, University of Melbourne, Parkville, Victoria 3052, Australia; 3 Aberdeen Oomycete Laboratory, College of Life Sciences and Medicine, University of Aberdeen–Foresterhill, Aberdeen, Scotland AB25 2ZD, UK; 4 Faculté de Médecine, Université Laval, Québec City, Québec, Canada G1V4G2; 5 Parasitology Section, Bernhard Nocht Institute for Tropical Medicine, Hamburg, Germany; 6 Department of Parasitology, Philipps University Marburg , Marburg 35043, Germany

## Abstract

*Plasmodium falciparum* exports proteins into erythrocytes using the *Plasmodium* export element (PEXEL) motif, which is cleaved in the endoplasmic reticulum (ER) by plasmepsin V (PMV). A recent study reported that phosphatidylinositol-3-phosphate (PI(3)P) concentrated in the ER binds to PEXEL motifs and is required for export independent of PMV, and that PEXEL motifs are functionally interchangeable with RxLR motifs of oomycete effectors. Here we show that the PEXEL does not bind PI(3)P, and that this lipid is not concentrated in the ER. We find that RxLR motifs cannot mediate export in *P. falciparum*. Parasites expressing a mutated version of KAHRP, with the PEXEL motif repositioned near the signal sequence, prevented PMV cleavage. This mutant possessed the putative PI(3)P-binding residues but is not exported. Reinstatement of PEXEL to its original location restores processing by PMV and export. These results challenge the PI(3)P hypothesis and provide evidence that PEXEL position is conserved for co-translational processing and export.

## Abstract (web summary)

Export of *Plasmodium falciparum* proteins into infected erythrocytes relies upon the PEXEL motif in target proteins. Here Boddey *et al.* challenge the hypothesis that the PEXEL motif mediates export by binding PI(3)P and instead suggest it acts via cleavage by plasmepsin V.

## Graphical abstract

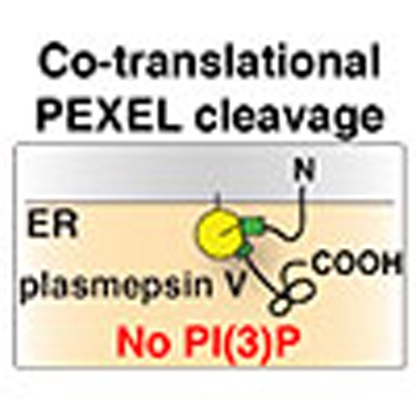

P. *falciparum* is a virulent parasite of humans. Following invasion of erythrocytes, the merozoite forms initiate remodelling, converting the terminally differentiated host cell into one in which the parasite can evade host responses and replicate (reviewed in ref. 1). Remodelling results in profound structural and morphological changes of the host cell central to parasite survival. This includes alterations of the cells physical properties, making it more rigid and gaining adhesive properties that can block flow in the microvasculature^2^,^3^. These parasite-induced changes of the erythrocyte play an important role in pathogenesis of malaria^4^.

The properties of *P. falciparum*-infected erythrocytes are conferred by export of hundreds of parasite proteins. To reach their destination, they traverse membranes of the parasite and parasitophorous vacuole (PV) and, in some cases, the erythrocyte membrane to reach the cell surface^5^. Consequently, the intracellular parasite exports many proteins establishing a trafficking network in the host cell. Most exported proteins contain a signal sequence for entry into the endoplasmic reticulum (ER) followed by 15–30 residues and a motif *Plasmodium* export element (PEXEL)/vacuolar transport signal^6^,^7^. The PEXEL is cleaved by the aspartyl protease plasmepsin V (PMV) in the ER, to uncover the export signal for trafficking to erythrocytes^1^,^8^,^9^,^10^,^11^. The PMV active site appears to be on the ER luminal side^12^. There are hundreds of proteins with a PEXEL predicted to be exported to the host erythrocyte^6^,^7^,^13^ via the *Plasmodium* translocon of exported proteins (PTEX) parasite translocon^14^,^15^.

Another group of exported proteins lack a PEXEL, the PEXEL-negative exported proteins^16^,^17^. This includes skeleton-binding protein 1^18^, membrane-associated histidine-rich proteins 1 and 2 (refs 19, 20), ring-exported proteins 1 and 2 (REX1 (ref. 21) and REX2 (ref. 22)), and the MSP-7-related proteins^17^, as well as *P. falciparum* erythrocyte membrane protein 1 (PfEMP1)^1^,^17^. PfEMP1 is trafficked to the surface of the erythrocyte where it adheres to host receptors on the microvasculature (reviewed in ref. 23). PEXEL-negative exported proteins are not cleaved by PMV and are selected for export through an unknown pathway but translocated via PTEX^14^,^15^.

Recently, a study reported phosphatidylinositol-3-phosphate (PI(3)P) binds PEXEL with high affinity and this phospholipid is concentrated on the ER lumenal membrane in *P. falciparum*^24^. In addition, PI(3)P binding in the ER, via the PEXEL Arg, is required for export independent of cleavage by PMV^24^. It was also reported that PEXEL is functionally interchangeable with RxLR of effector proteins from oomycetes that are translocated into host cells^24^,^25^. Owing to the implications of these findings, our six laboratories have examined key findings of Bhattacharjee *et al.*^24^,^25^. In contrast, we find PEXEL does not bind PI(3)P, this phospholipid is not concentrated in the ER of *P. falciparum* but on the food vacuole and apicoplast membranes, that the PEXEL is not functionally interchangeable with the RxLR motif of oomycetes and the PEXEL is located at a conserved location to ensure co-translational processing by PMV for export. Therefore, PI(3)P binding of PEXEL in the ER is not the selective mechanism for export of malarial effector proteins to the *P. falciparum*-infected erythrocyte.

## Results

### *P. falciparum* exported proteins do not bind PI(3)P

**Figure 1:**
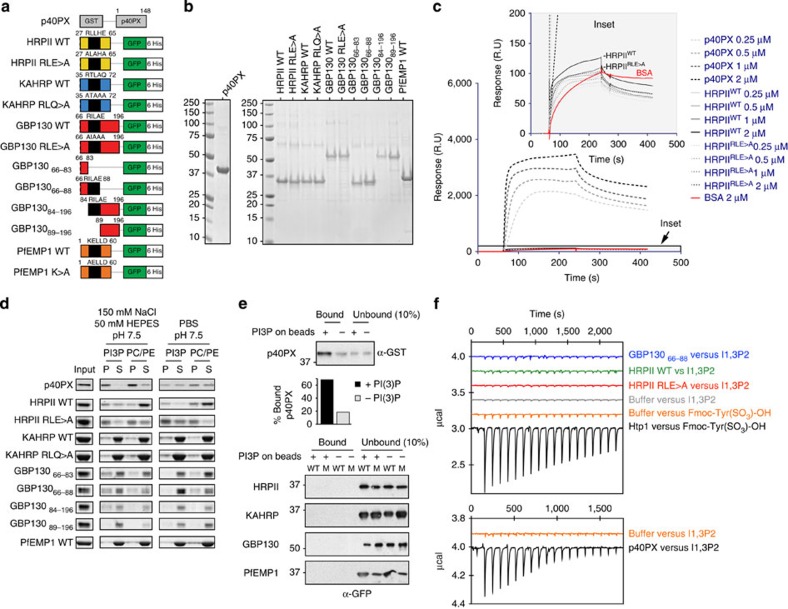
Exported *P. falciparum* proteins do not bind PI(3)P. (**a**) Recombinant proteins expressed in this study as fusions to GST or GFP/6His. (**b**) Recombinant proteins stained with Coomassie blue stain. Molecular weights (kDa) are shown. (**c**) Binding of p40PX and HRPII with native PEXEL (WT) or mutant PEXEL (RLE>A) to PI(3)P liposomes measured by SPR. Binding to control PC/PE liposomes was subtracted to give the sensorgrams^24^. BSA was used to measure nonspecific binding to the liposome-coated chip (red). Inset: a similar level of low binding of HRPII^WT^ and HRPII^RLE>A^ to PI(3)P liposomes. Experiments were performed in triplicate. (**d**) Binding of proteins to PI(3)P liposomes or control PC/PE liposomes, determined by ultracentrifugation. Pellet and supernatant fractions were resolved by SDS–PAGE and Coomassie blue stain. Proteins were ultracentrifuged in buffer to remove potential aggregates before incubation with liposomes^39^, explaining why input and sum of pellet and supernatant sometimes differ. Experiments were performed in triplicate. Full-length gels shown in Supplementary Fig. 6. (**e**) Binding of p40PX and *P. falciparum* proteins with native PEXEL (WT) or mutant PEXEL (M) to PI(3)P-coated beads (+) or control beads lacking lipid (−). Bound protein was eluted in SDS–PAGE sample buffer and detected by immunoblot with anti-GST (p40PX) or anti-GFP (*P. falciparum* proteins). Ten per cent of unbound fraction volume was loaded to visualize protein inputs. Densitometry (histogram shown) of the p40PX bands shows that 68% of p40PX input bound to PI(3)P-coated beads (PI3P+) compared with 18% of input to control beads (PI3P−). No binding was detected for *P. falciparum* exported proteins. Experiments were performed in triplicate. Full-length gels shown in Supplementary Fig. 7. (**f**) Binding of recombinant proteins to phospholipid head group of PI(3)P (inositol 1,3 bisphosphate; I1,3P2) in solution, measured by isothermal calorimetry. The titration did not differ between I1,3P2 (2.4 mM) into dialysis buffer (150 mM NaCl, 20 mM HEPES pH 7.4) (negative control) and the same buffer containing either GBP130_66–88_, HRPII^WT^ or HRPII^RLE>A^. As controls, binding of Htp1 from *S. parasitica* to Fmoc-Tyr(SO_3_)-OH and also p40PX binding to I1,3P2 are shown. Experiments were performed in duplicate.

It has been suggested that PI(3)P specifically binds PEXEL of exported proteins^24^. This hypothesis was tested using proteins containing a PEXEL, or with this motif mutated. The amino terminus of exported proteins, histidine-rich protein II (HRPII)^7^,^26^, knob-associated histidine-rich protein (KAHRP)^6^ and glycophorin binding protein 130 (GBP130)^7^,^26^, which all contain a wild-type or mutant PEXEL motif, were expressed as green fluorescent protein (GFP)/6His fusions (Fig. 1a,b). The N terminus of PfEMP1, which does not have a PEXEL, has also been shown to bind PI(3)P^24^ and it was expressed fused to GFP/6His (Fig. 1a,b). The p40PX protein, which contains a PX domain that specifically binds PI(3)P, was used as a positive control^24^.

To determine whether p40PX bound PI(3)P, surface plasmon resonance (SPR) was performed following methods of Battacharjee *et al.*^24^. Proteins were flowed over a chip coated with phosphatidylcholine (PC):phosphatidylethanolamine (PE):C_16_PI(3)P (75:20:5) liposomes (Fig. 1c). As expected, p40PX bound quantitatively to liposomes (*K*_d_: 195 nM). HRPII^WT^ showed very little binding and the control protein HRPII^RLE>A^, in which the PEXEL had been mutated, showed similarly low binding, demonstrating PEXEL of HRPII does not mediate specific binding. Both proteins bound with a similar response to background binding of BSA (Fig. 1c). HRPII–GFP proteins were fluorescent, indicating they were folded, and gel filtration analysis confirmed they were monomers (Supplementary Fig. 1a). Similarly, KAHRP^WT^, GBP130^WT^, KAHRP^RLQ>A^ and GBP130^RLE>A^ binding was too weak to quantify (Supplementary Fig. 1b). Although the purchased p40PX bound quantitatively to PI(3)P (*K*_d_: 196 μM), the Req was less than p40PX expressed and purified by ourselves (Fig. 1c). This demonstrated that proteins expressed and purified by our laboratories could bind PI(3)P strongly; however, neither the PEXEL motif nor the surrounding sequences of exported *P. falciparum* proteins produced specific binding to PI(3)P above background.

As we could not detect specific PEXEL binding to PI(3)P by SPR, we tested other formats. Liposomes containing either PI(3)P or control (PC/PE) were tested for binding in solution^24^ and proteins sampled by ultracentrifugation. p40PX protein bound strongly to PI(3)P liposomes in HEPES buffer and could be blocked using PBS (phosphate competes with phosphorylated lipids for lipid binding). Some p40PX could be detected in the pellet of control liposomes, suggesting PI(3)P-independent binding (Fig. 1d). Nevertheless, KAHRP^WT^, KAHRP^RLQ>A^, GBP130_66–83_, GBP130_66–88_, GBP130_84–196_, GBP130_89–196_ and PfEMP1^WT^ showed no PEXEL-specific binding. HRPII^WT^ showed binding to PI(3)P and PC/PE liposomes but the HRPII protein, in which the PEXEL was mutated (HRPII^RLE>A^), bound at a similar level and PBS had no effect, providing strong evidence this motif was not responsible for binding. Our results demonstrated no correlation between the presence of PEXEL and specific binding to PI(3)P liposomes, suggesting they do not specifically interact.

To further test whether HRPII^WT^, HRPII^RLE>A^, KAHRP^WT^, KAHRP^RLQ>A^, GBP130^WT^, GBP130^RLE>A^ and PfEMP1^WT^ bound PI(3)P, they were incubated with PI(3)P-coated beads or controls. The p40PX protein bound strongly to PI(3)P (68% bound) but the *P. falciparum* proteins showed no binding (Fig. 1e). Similar results were obtained with GBP130_66–83_, GBP130_66–88_, GBP130_84–196_ and GBP130_89–196_, confirming these proteins did not bind PI(3)P (Supplementary Fig. 2a).

To analyse whether PEXEL could bind PI(3)P in solution, isothermal titration calorimetry (ITC) was performed with HRPII^WT^, HRPII^RLE>A^, GBP130_66–88_ and GBP130_66–196_ using the head group of PI(3)P, inositol 1,3 diphosphate (I1,3P_2_). The proteins did not show any thermal interaction signature with I1,3P_2_ that was indicative of binding (Fig. 1f and Supplementary Fig. 2c). This was not due to instrument sensitivity, as titrations were performed between Htp1 from *Saprolegnia parasitica* (SpHtp1^24-198^(His)_6_) and the ligand Fmoc-Tyr(SO_3_)-OH and between p40PX and I1,3P_2_, and binding interactions were observed for these proteins (Fig. 1f)^24^,^27^.

Finally, lipid binding was assessed using spot membranes as described previously^28^. GBP130_66–196_ and GBP130_66–196_RLE>A containing a mutated PEXEL showed binding to PI(3)P, PI(4)P and PI(5)P on membranes; however, the PEXEL mutant also bound, suggesting it was not responsible for the interaction (Supplementary Fig. 2d). Binding to lipid spot membranes can occur by a small proportion of misfolded proteins and the interaction blocked by sub-stoichiometric amounts of DnaK, a chaperone that specifically binds misfolded proteins^28^. To test whether GBP130 proteins were binding lipids in this way, DnaK was incubated with GBP130_66–196_ and this blocked binding (Supplementary Fig. 2e), confirming PEXEL of GBP130 does not specifically interact with PI(3)P, and that misfolded PEXEL proteins interact with lipids.

In summary, we did not detect specific binding of HRPII, KAHRP, GBP130 or PfEMP1 to PI(3)P using methods published previously^24^, as well as alternative approaches.

### PEXEL and RxLR proteins do not bind PI(3)P

It was reported that PEXEL protein HRPII binds PI(3)P in the *P. falciparum* ER before cleavage by PMV, as determined by immunoprecipitation and measuring bound lipid by enzyme-linked immunosorbent assay (ELISA)^24^. We followed the same protocol using proteins, to measure bound PI(3)P. We immunoprecipitated (IP) proteins that either had a PEXEL and were cleaved and exported or contained mutations that blocked cleavage and export. We expected PEXEL-cleaved proteins would not co-IP PI(3)P, owing to rapid removal of lipid-binding residues as reported (that is, conserved PEXEL Arg)^24^; however, if the PI(3)P-binding hypothesis was correct, that proteins containing such N-terminal lipid-binding residues such as KAHRPΔ38–48 (containing RTLAQ), HRPIIss-AVR3a (containing RLLR) and KAHRP R>K (containing KTLAQ; the PEXEL-like lysine residue in PfEMP1 previously reported to bind PI(3)P^24^), would co-IP PI(3)P. However, although PI(3)P ELISA controls were detected, allowing the generation of a standard curve, the quantity of PI(3)P on purified chimeras was minimal and no pattern was evident between PEXEL/RxLR-containing and PEXEL mutant proteins (Supplementary Fig. 3a). One possibility for this discrepancy is Bhattacharjee *et al.*^24^ used HIV protease inhibitor lopinavir to inhibit PMV, to retain more uncleaved HRPII precursor before IP. Lopinavir is a poor PMV inhibitor and lethal to *P. falciparum*, probably acting on other aspartyl proteases^10^. To test whether lopinavir affects viability under the conditions described^24^, we treated parasites and assessed translation. This showed lopinavir had a more potent effect on inhibiting translation than antimalarials, strongly suggesting the parasites were dying (Supplementary Fig. 3b). Under these conditions, it is possible that Bhatachaerjee *et al.*^24^ detected anomalous lipid binding to their reporters.

### PI(3)P is present on the food vacuole membrane

**Figure 2:**
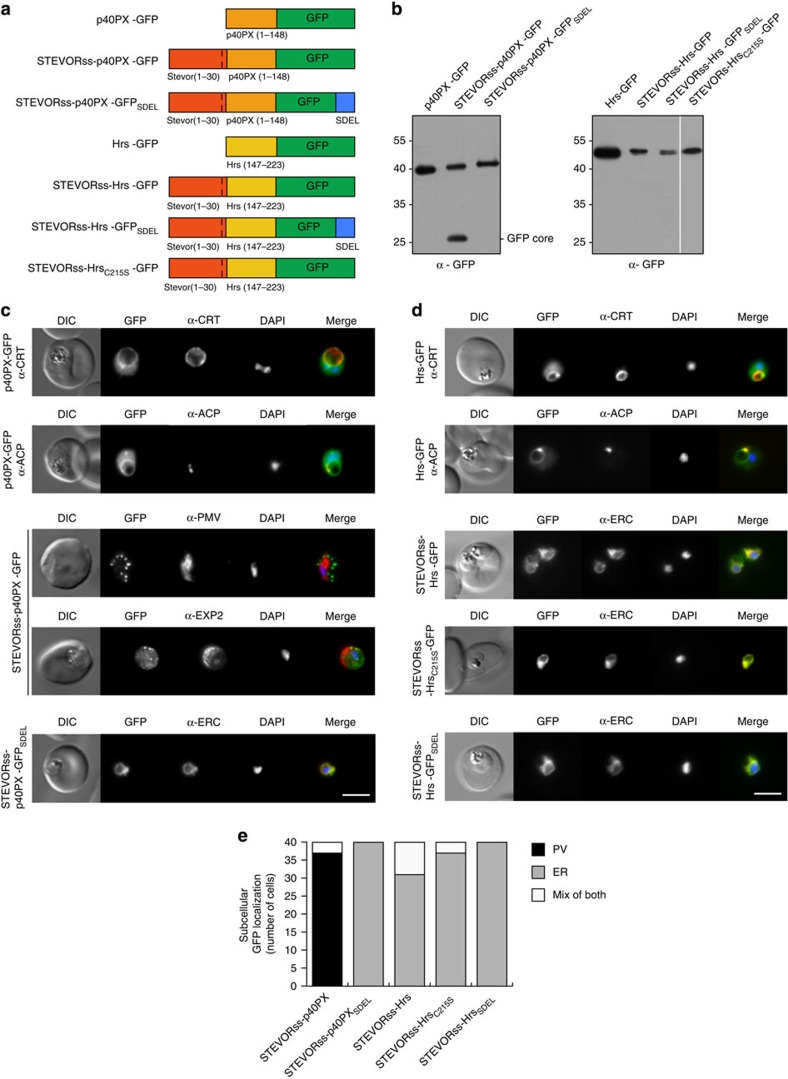
PI(3)P is not localized in the ER of *P. falciparum*. (**a**) Chimeric proteins expressed in *P. falciparum*. p40PX-GFP, no signal sequence; STEVORss-p40PX-GFP, STEVOR signal sequence; STEVORss-p40PX-GFP_SDEL_, STEVOR signal sequence and ER retention signal; Hrs-GFP, no signal sequence; STEVORss-Hrs-GFP, STEVOR signal sequence; STEVORss-Hrs_SDEL_-GFP, signal sequence and ER retention signal; STEVORss-Hrs_C215S_-GFP, STEVOR signal sequence and mutation of lipid-binding residue C215S in both FYVE fingers. (**b**) p40PX and Hrs reporters are expressed in *P. falciparum*-infected erythrocytes. Blots probed with anti-GFP antibodies. First panel: p40PX-GFP, STEVORss-p40PX-GFP and STEVORss-p40PX-GFP_SDEL._ A ‘GFP core' band was observed for STEVORss-p40PX-GFP. This is observed for all GFP chimeras secreted to the PV and does not indicate degradation specific to this protein. Note the absence of ‘GFP core' when SDEL ER-retention signal was attached, which prevented secretion to PV (lane 3). Second panel: Hrs-GFP, STEVORss-Hrs-GFP, STEVORss-Hrs-GFP_SDEL_ and STEVORss-HrsC_215S_-GFP. Note the lack of ‘GFP core' for STEVORss-Hrs fusions, confirming they were not secreted. (**c**) p40PX-GFP localizes to the cytoplasm and to membranes of the food vacuole (labelled with anti-CRT) and apicoplast (labelled with anti-ACP). STEVORss-p40PX-GFP localizes to the PV in *P. falciparum*-infected erythrocytes, as shown by co-localization with anti-EXP2, and not in the ER, which was labelled with anti-PMV. If PI(3)P was located within the ER, this chimera would be expected to remain within the ER via p40PX binding. Fusion to an ER-retention signal (STEVORss-p40PX-GFP_SDEL_) retains the protein in ER, co-localization with anti-ERC. Scale bar, 5 μm. (**d**) Localization of Hrs-GFP to the cytoplasm and membranes of food vacuole (labelled anti-CRT) and apicoplast (labelled anti-ACP). STEVORss-Hrs-GFP localizes to the ER as shown by co-localization with anti-ERC; however, mutation of residues necessary for lipid binding in both Hrs FYVE fingers (STEVORss-Hrs_C215S_-GFP) does not abrogate ER localization, demonstrating PI(3)P-independent retention in ER. Addition of ER retention signal (STEVORss-Hrs-GFP_SDEL_) also causes ER localization. Scale bar, 5 μm. (**e**) Quantification of GFP localization in the PV (black), ER (grey), or mix of both (white) determined using 40 *P*. *falciparum*-infected erythrocytes per construct (20 cells per experiment, performed twice).

Bhattacharjee *et al.*^24^ localized PI(3)P to the inner leaflet of ER membranes in *P. falciparum* by expressing fluorescent reporters fused to PI(3)P-binding domains of two endosomal proteins, p40-phox-PX and early endosomal antigen 1 (EEA1)^24^. To localize PI(3)P, we made transgenic parasites expressing p40PX fused to GFP (p40PX-GFP) in different parasite subcellular compartments (Fig. 2a,b). The p40PX-GFP protein, lacking a signal sequence, was expressed in the cytoplasm and the fluorescence was concentrated around the food vacuole, as determined by co-localization with *P. falciparum* chloroquine resistance transporter^29^, and the apicoplast, as determined by co-localization with acyl carrier protein (ACP) (Fig. 2c). This was consistent with previous localization of PI(3)P at the food vacuole and apicoplast^30^, and the lysosomal membrane and endocytic pathway in other organisms^31^. To determine whether PI(3)P was present on the luminal side of ER membranes, we fused p40PX-GFP to a signal sequence from STEVOR (STEVORss-p40PX-GFP) (Fig. 2c)^32^. This targeted the protein into the ER, where it would be retained bound to PI(3)P as reported^24^. However, a circumferential localization around the parasite was observed, indicating it was secreted to the parasite periphery and PV. This was confirmed by the presence of a ‘GFP core' species by immunoblotting (Fig. 2b), which results from endocytosis from the PV to the food vacuole during haemoglobin uptake; this confirms the chimera was secreted and not ER retained. Experiments using antibodies to the ER resident protease PMV^33^ failed to show co-localization with GFP, indicating the reporter was not retained in the ER (Fig. 2c,e)^24^. The GFP localization was similar to EXP2 (ref. 34), a marker of the PV confirming secretion. To exclude the possibility that the STEVOR signal sequence influenced this localization, we fused p40PX-GFP to the ACP signal sequence and it trafficked to the PV and was not retained in the ER (Supplementary Fig. 4). This PV localization was also observed when the PI(3)P-binding residue of p40PX was mutated (R58Q, Supplementary Fig. 4). This suggests p40PX did not bind to the inner leaflet of the ER, consistent with a lack of PI(3)P. Only when an SDEL retention signal^35^ was fused to the carboxy terminus of STEVORss-p40PX-GFP did it accumulate in the ER, as shown by co-localization with the ER calcium-binding protein (ERC) (Fig. 2c,e). Therefore, PI(3)P is present on the food vacuole and apicoplast membranes of *P. falciparum* but not within the ER^24^.

To further assess the location of PI(3)P in *P. falciparum*, we expressed Hrs with two FYVE fingers^36^, in different subcellular compartments of the parasite, using a STEVOR signal peptide and GFP (Fig. 2a,b). When Hrs2xFYVE was expressed in the cytoplasm, Hrs-GFP was concentrated around the food vacuole and apicoplast, consistent with PI(3)P at these membranes (Fig. 2d). However, when it was directed through the ER (STEVORss-Hrs-GFP), the protein co-localized with the ER marker, ERC, similar to when an ER retention signal was added (Fig. 2d,e), and was not secreted to the PV (note the absence of ‘GFP core' Fig. 2b). However, when residues needed for PI(3)P binding were mutated, the protein remained in the ER. Therefore, ER retention observed for STEVORss-Hrs-GFP and STEVORss-HRS_C215S_-GFP was not due to PI(3)P-specific binding, suggesting another interaction(s) was retaining reporters in the ER, possibly involving unfolded proteins and ER chaperones. These results are consistent with the lack of PI(3)P in the parasite ER.

### The RxLR motif cannot mediate export in *P. falciparum*

**Figure 3:**
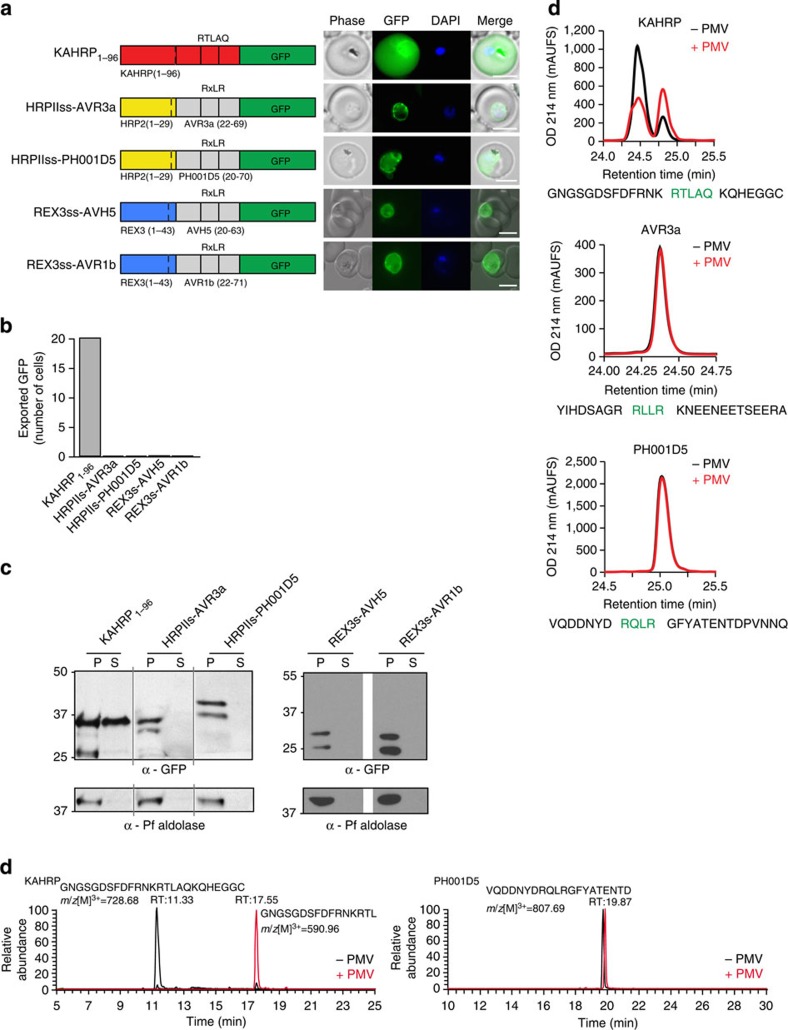
Oomycete RxLR does not mediate export in *P. falciparum*. (**a**) KAHRP_1–96_ is exported to the *P. falciparum*-infected erythrocyte; however, HRPIIss-AVR3a, HRPIIss-PH001D5, REX3ss-AVH5 and REX3ss-AVR1b are not exported but accumulate in the parasite and PV. Scale bar, 5 μm. (**b**) Quantification of cells with exported GFP determined using 20 *P*. *falciparum*-infected erythrocytes per construct (10 cells per experiment, performed twice). (**c**) Immunoblot with anti-GFP antibodies of each chimera from the tetanolysin pellet (P) and supernatant (S) shows that KAHRP_1–96_ is exported but RxLR chimeras are not. Tetanolysin inserts pores in the erythrocyte membrane, allowing sampling of the host cell cytosol. Aldolase was included as a control, as described previously^71^. Full-length gels shown in Supplementary Fig. 8. (**d**) RP-HPLC shows the KAHRP peptide is cleaved when incubated with PMV (red). AVR3a and PH001D5 peptides are not cleaved by PMV. PEXEL/RxLR sequences are labelled green. (**e**) MS/MS-extracted ion chromatograms of KAHRP and PH001D5 peptides after incubation with PMV shows that KAHRP peptides were cleaved after the PEXEL Leu (GNGSGDSFDFRNKRTL−) but PH001D5 was not cleaved.

The RxLR amino acid motif in effectors of oomycetes has been implicated in export to host cells^37^. The RxLR motif from *Phytophthora infestans* also reportedly allows export from *P. falciparum* and *Plasmodium berghei* into erythrocytes^24^,^25^. Expression of PEXEL in *P. infestans* proteins may also aid in translocation of chimeric effector proteins^38^,^39^. This suggested RxLR was functionally transferable with PEXEL in *Plasmodium* spp. We therefore tested whether RxLR could mediate export in *P. falciparum* and if it was a substrate for PMV, as is the PEXEL. To examine this, we used the same constructs reported by Bhattacharjee *et al.*^25^, where the signal sequence of *P. falciparum* HRPII was fused to 47 or 49 amino acids of the *P. infestans* proteins, Avirulence protein 3a (AVR3a) or PH001D5, respectively, which contained RxLR in an equivalent position as PEXEL in *P. falciparum*^6^,^7^,^40^. These were fused to GFP (HRPIIss-AVR3a and HRPIIss-PH001D5) and expressed in *P. falciparum*. Both proteins were retained in the parasite and PV, and not exported, in contrast to KAHRP_1–96_, which contains a PEXEL and was exported (Fig. 3a,b). To further examine RxLR, we made two constructs where the signal sequence of REX3, a *P. falciparum* exported protein, was fused to the RxLR region of *Phytophthora sojae* AVH5 (REX3ss-AVH5)^41^ or AVR1b (REX3ss-AVR1b)^42^ followed by GFP. When expressed in *P. falciparum*, REX3ss-AVH5 and REX3ss-AVR1b entered the ER and were trafficked to the PV, but not exported (Fig. 3a,b). These data show RxLR from different *Phytophthora* spp. cannot mediate export to the host cell by *P. falciparum*.

Export of PEXEL proteins requires cleavage by PMV^10^,^11^,^43^. This was confirmed using KAHRP_1–96_, which was exported into the erythrocyte (Fig. 3a) and the cleaved protein detected in the tetanolysin supernatant (Fig. 3c). Tetanolysin inserts pores in the erythrocyte membrane, allowing sampling of proteins in the host cell cytosol^44^. Cleavage of KAHRP by PMV in *P. falciparum* occurs after the PEXEL Leu^9^. In contrast, HRPIIss-AVR3a, HRPIIss-PH001D5, REX3ss-AVH5 and REX3ss-AVR1b showed no signal in the tetanolysin supernatant, despite evidence of some N-terminal processing, demonstrating these proteins are not exported nor cleaved in a manner competent for export (Fig. 3c). To understand the mechanism, we examined whether RxLR is a substrate for PMV (Fig. 3d,e). Peptides containing RxLR from AVR3a or PH001D5 were incubated with PMV and analysed by mass spectrometry^10^. In contrast to KAHRP peptides that were cleaved after the PEXEL Leu by PMV, neither AVR3a nor PH001D5 peptides were affected. To confirm this, we affinity purified HRPIIss-PH001D5 from *P. falciparum*-infected erythrocytes and examined the proteins by mass spectrometry (MS). We detected peptides N-terminal to RxLR and the intact peptide as well (Supplementary Fig. 5). Thus, RxLR is not a substrate for PMV, consistent with it not mediating export in *P. falciparum*.

### Export of PEXEL proteins is not independent of PMV

**Figure 4:**
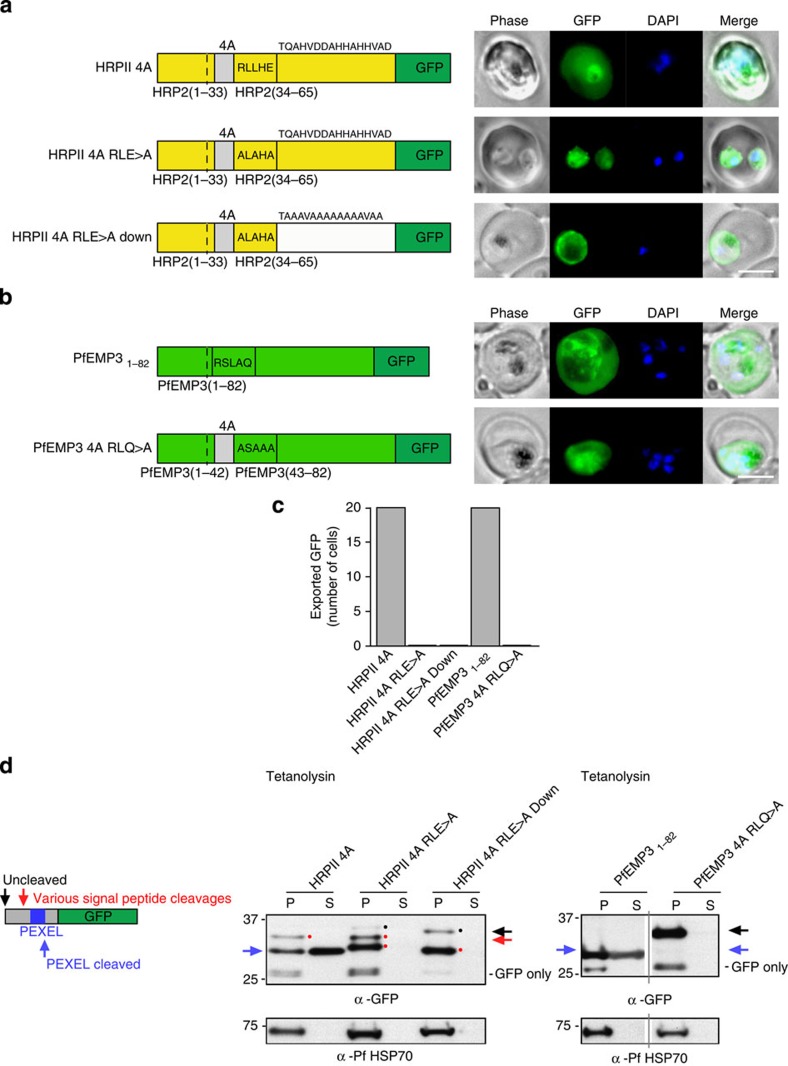
PEXEL-independent export of PEXEL proteins does not occur in *P. falciparum*. (**a**) HRPII 4A is exported to the *P. falciparum-*infected erythrocyte. HRPII 4A RLE>A and HRPII 4A RLE>A Down are not exported. Scale bar, 5 μm. (**b**) PfEMP3_1–82_ is exported by *P. falciparum* to the erythrocyte, but PfEMP3 4A RLQ>A is not. Scale bar, 5 μm. (**c**) Quantification of cells with exported GFP determined using 20 *P. falciparum*-infected erythrocytes per construct (10 cells per experiment, performed twice). (**d**) Immunoblot with anti-GFP antibodies of chimeras from the tetanolysin pellet (P) and supernatant (S) shows that HRPII 4A and PfEMP3_1–82_ are cleaved at the PEXEL (blue arrow) and exported. Signal peptide-cleaved HRPII 4A was also visible (red spot). HRPII 4A RLE>A, HRPII 4A RLE>A Down and PfEMP3_1–82_ RLQ>A were not exported and bands corresponding to signal peptide-cleaved (various positions possibly due to insertion of four alanines; red spots/arrow) and uncleaved protein (black spots/arrow) are present. Full-length gels shown in Supplementary Fig. 9.

It was reported that PEXEL proteins HRPII and PfEMP3 can be exported via an alternative mechanism if PEXEL is mutated when four alanines are inserted immediately after the signal sequence^24^,^45^. To test this, we generated similar constructs^24^,^45^ of HRPII with a signal sequence and PEXEL (RLLHE) fused to GFP having four alanines inserted immediately after the signal sequence (Supplementary Table 1). HRPII 4A had a PEXEL and was exported (Fig. 4a). However, when the PEXEL was mutated (HRPII 4A RLE>A), no export was observed and it accumulated in the parasite and PV. Mutation of residues C-terminal to the mutant PEXEL (HRPII 4A RLE>A Down)^24^,^45^ did not allow export (Fig. 4a,b). The HRPII proteins were analysed by tetanolysin fractionation and immunoblotting. The HRPII 4A protein with a PEXEL was present in the supernatant fraction and processed by PMV, confirming it was exported (Fig. 4d). However, both HRPII 4A RLE>A and HRPII 4A RLE>A proteins were absent from supernatant fractions and not exported, despite evidence of some processing N-terminal to the mutant PEXEL sequence (Fig. 4d). The lack of export of HRPII 4A RLE>A was in contrast to previous work with this construct and shows cleavage by PMV is required^24^.

Similarly, PfEMP3_1–82_ with a PEXEL was exported (Fig. 4b,c) and processed by PMV (Fig. 4d). Cleavage of PfEMP3 by PMV in *P. falciparum* occurs after the PEXEL Leu^10^. However, mutation of PEXEL RLQ>A blocked cleavage by PMV and resulted in no export, including when four alanines were fused immediately after the signal sequence (Fig. 4b–d). In contrast to earlier work^24^,^45^, these results with HRPII and PfEMP3 demonstrate export occurs only when PEXEL was present and processed by PMV.

### Spatial positioning of the PEXEL is important for export

**Figure 5:**
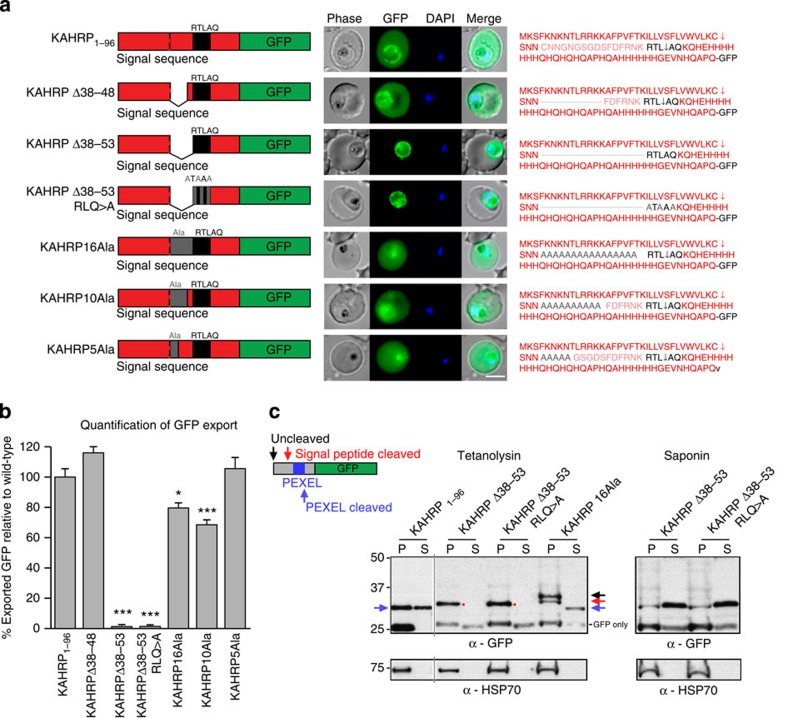
Correct PEXEL positioning is essential for export. (**a**) KAHRP_1–96_ and KAHRPΔ38–48 are exported to the *P. falciparum*-infected erythrocyte but KAHRPΔ38–53 and KAHRPΔ38–53 RLQ>A accumulate in the PV and are not exported. KAHRP16Ala, KAHRP10Ala and KAHRP5Ala are exported to the red blood cell. Scale bar, 5 μm. (**b**) Quantification of export in **a**. Data are the mean (±s.e.m.) GFP fluorescence intensity in the host cell from 20 cells per construct (20 replicates), shown as a percentage of KAHRP_1–96_. Data were analysed by *t*-test (***P<0.0001 and *P<0.003, compared with KAHRP_1–96_). (**c**) Immunoblot with anti-GFP antibodies of chimeras from the tetanolysin and saponin pellet (P) and supernatant (S). This shows KAHRP_1–96_ at a size corresponding to cleavage at the PEXEL and it was exported (32 kDa; blue arrows), KAHRPΔ38–53 and KAHRPΔ38–53 RLQ>E were cleaved to a size corresponding to cleavage by signal peptidase (32.5 kDa; red spots) and were not exported, but were present in the saponin supernatant, confirming they were secreted to the PV, and that KAHRP16Ala was of a size corresponding to cleavage at the PEXEL and it was exported (32 kDa; blue arrow); however, cleavage was inefficient as bands consistent with signal sequence cleaved (red arrow) and uncleaved (black) species were present in the pellet fraction. HSP70 was used as a control. Full-length gels shown in Supplementary Fig. 10.

It has been reported that PEXEL proteins can be exported independent of PMV activity by binding PI(3)P. We sought to generate a protein containing a PEXEL not cleaved by PMV, to assess whether it could be exported by an alternative mechanism such as PI(3)P. The PEXEL is located at a conserved position in exported proteins (15–30 amino acids C-terminal to the signal sequence)^13^ and we investigated the effect of shifting this position towards the N-terminal signal sequence. KAHRP_1–96_ containing a PEXEL in the native position was efficiently exported (Fig. 5a,b). Deletion of 11 amino acids N-terminal to the PEXEL (KAHRPΔ38–48) had no negative effect on export, consistent with these amino acids having no essential role (Fig. 5a,b). However, deletion of 16 amino acids abutting the PEXEL (KAHRPΔ38–53), which placed the motif 3 residues beyond the signal sequence cleavage site (LKC—SNN), determined previously for KAHRP^9^, blocked export and the protein was secreted to the PV (Fig. 5a,b). The same PV localization was observed when PEXEL was mutated (KAHRPΔ38–53RLQ>A). Reinstatement of PEXEL to its native position using a 16 alanine spacer (16Ala) restored export, showing that motif positioning, and not primary amino acid sequence N-terminal to the PEXEL, is critical for export. Incorporation of six residues of KAHRP immediately abutting PEXEL (10Ala) did not improve export above 80% of controls; however, inclusion of 11 KAHRP residues abutting PEXEL (5Ala) fully restored export. It is possible that residues in this area assist export by improving the affinity of the substrate for PMV, although not being critical for processing.

To validate export of KHARP proteins, tetanolysin and saponin fractionation was performed (Fig. 5c). KHARP_1–96_ was present in the tetanolysin supernatant and efficiently processed by PMV. In comparison, KAHRPΔ38–53 and KAHRPΔ38–53RLQ>A were absent from the tetanolysin supernatant but present in the saponin supernatant, confirming their localization in the PV but not the erythrocyte. Their identical localization and indistinguishable molecular weight was consistent with cleavage by signal peptidase (Fig. 5a,c). Conversely, KAHRP16Ala was present as a PMV-cleaved protein in the tetanolysin supernatant, confirming it was exported; however, the presence of higher bands in pellet fractions, which correspond to uncleaved and signal peptidase-cleaved forms, determined previously^9^ indicate the 16Ala mutant form was cleaved inefficiently by PMV, which may explain why export was slightly less than the control (Fig. 5a,b). Collectively, these results show spatial position of PEXEL at the N terminus of KAHRP is important for export and linked to efficiency of cleavage by PMV. Further studies are needed to confirm this with other exported PEXEL proteins.

### Co-translational PEXEL processing occurs before export

**Figure 6:**
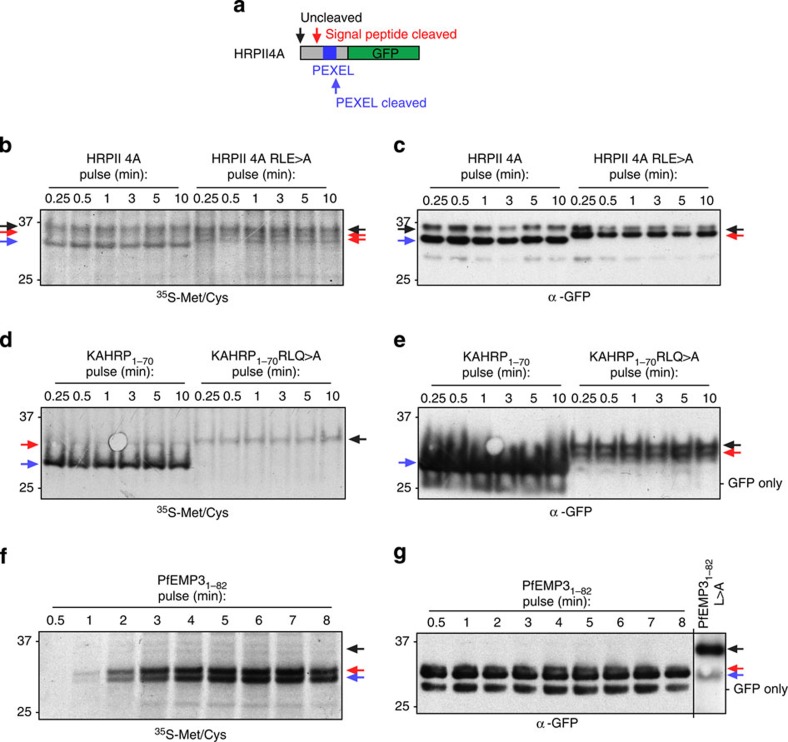
The PEXEL is cleaved co-translationally. (**a**) Top: schematic of PEXEL proteins and their cleavage positions. (**b**) ^35^S-methionine/cysteine-labelling (^35^S-Met/Cys) of HRPII 4A and HRPII 4A RLE>A in *P. falciparum* for the indicated times shows bands corresponding to uncleaved (black arrow), signal peptide-cleaved (red arrow) and PEXEL-cleaved (blue arrow) species. Two signal peptide-cleaved species are present for RLE>A (red arrows), possibly due to insertion of four alanines following the signal sequence. (**c**) Immunoblot with anti-GFP antibodies of the same membrane shows the bands are GFP specific. (**d**) ^35^S-Met/Cys-labelling of KAHRP_1–70_ and KAHRP_1–70_-RLQ>A in *P. falciparum*. Bands corresponding to uncleaved (black arrow), signal peptide-cleaved (red arrow) and PEXEL-cleaved (blue arrow) species are indicated. (**e**) Immunoblot with anti-GFP antibodies of the same membrane. Note the absence of a signal peptidase cleaved product for KAHRP_1–70_ RLQ>A in the autoradiography that is present in the immunoblot as the latter reveals protein produced over hours. This shows signal peptidase activity was inefficient. (**f**) ^35^S-Met/Cys-labelling of PfEMP3_1–82_ in *P. falciparum*. Bands corresponding to cleavage of the indented signal peptide (red arrow) and PEXEL (blue arrow) are indicated. (**g**) Immunoblot with anti-GFP antibodies of the same membrane. A PEXEL mutant (PfEMP3_1–82_ L>A) was included as a size control. Most protein detected with anti-GFP was present before radiolabelling; thus, quantity is not comparable to ^35^S-Met/Cys. Incorporation of ^35^S-Met/Cys into HRPII and KAHRP constructs did not increase appreciably over time, in contrast to PfEMP3. The reason for this is unknown but may be due to saturation of anti-GFP agarose in the former experiments, which was used to enrich the GFP proteins. Full-length gels for all panels shown in Supplementary Fig. 11.

A paradox of the PI(3)P hypothesis is that the reported lipid-binding residues of PEXEL^24^ are removed by PMV in the ER. We investigated the dynamics of PEXEL cleavage to measure the rate of processing by labelling with ^35^S-methionine/cysteine. HRPII 4A (Fig. 6a) showed three bands of a size corresponding to uncleaved protein and the signal peptidase and PMV cleavage products just 0.25 min after addition of labelled amino acids (Fig. 6b,c). Mutation of the PEXEL RLE>A blocked its cleavage, while the uncleaved and the signal peptidase-cleaved bands were still observed, as well as an additional band that probably corresponds to a second signal peptidase-cleaved species derived from cleavage at the four alanines after the signal peptide. Similarly, for KAHRP_1–70_ there was rapid appearance of the PEXEL-cleaved protein, which was blocked by mutation of the motif RLQ>A (Fig. 6d,e). The KAHRP_1–70_RLQ>A construct was inefficiently cleaved by signal peptidase (cleavage of KAHRP by signal peptidase occurs at ^32^LKC↓SNN^37^ and this has been confirmed by MS^9^) and did not accumulate, raising the possibility that this protein may not efficiently enter the ER, resulting in reduced protein levels either by stalled translation or proteosomal degradation. This was supported by the absence of signal peptidase-cleaved protein in the 35S blot, which was however detected by immunoblot as this methodology detects protein produced over hours. KAHRP RLQ>A has been shown previously to be cleaved by signal peptidase inefficiently; however, a species that had the signal peptide removed was secreted to the PV^9^. Pulse-chase experiments with PfEMP3_1–82_ also demonstrated that cleavage by signal peptidase and PMV occurred concomitantly with translation, although the product of PMV cleavage was consistently less abundant, raising the possibility this protein may be less co-translationally cleaved by PMV than HRPII and KAHRP (Fig. 6f,g)^43^. The rapid appearance of PEXEL-cleaved and signal sequence-cleaved species of HRPII, KAHRP and PfEMP3 suggest they are co-translationally processed by PMV and signal peptidase at the ER membrane. Thus, if the PEXEL was available for binding to PI(3)P for export, the Arg residue involved in binding^24^ would be very rapidly removed by PMV.

## Discussion

Bhattacharjee *et al.*^24^,^25^,^45^ have suggested the PEXEL directs export by binding to PI(3)P on the inner ER leaflet, and that the RxLR motif of *P. infestans* can functionally replace PEXEL. They also showed RxLR was not cleaved by PMV and concluded export of PEXEL proteins occurs independent of this enzyme. In this study, we show PEXEL proteins and PfEMP1 do not bind PI(3)P, that this lipid is not present inside the ER, and that RxLR cannot functionally complement the PEXEL in *P. falciparum*. Thus, we find no evidence for PI(3)P in recognition of proteins in the ER for export. Rather, it is evident that *P. falciparum* has evolved PEXEL at a conserved spatial position ensuring co-translational cleavage by PMV at the ER for export.

Consistent with our findings, PMV activity previously has been shown to be essential for export and parasite survival. Expression of catalytic PMV mutants in independent laboratories gave a dominant-negative phenotype that reduced export and slowed parasite growth^11^,^43^. A similar but stronger effect on export and parasite lethality was observed using transition-state PEXEL mimetics that specifically inhibit PMV^43^,^46^. The function of PMV has also been determined showing it cleaves PEXEL proteins in the ER at the Leu residue, to expose an export signal necessary for the next step^10^,^11^. PEXEL appears to be cleaved rapidly, as we could not label uncleaved products even using short time points. Cleavage by signal peptidase was also observed at the earliest time points, suggesting both enzymes act co-translationally at the ER membrane. Although this model is based on the likelihood that the PMV catalytic domain is in the ER lumen, we cannot completely exclude it associates with the cytoplasmic face and PEXEL cleavage occurs on this side^47^,^48^. This would require that proteins are assembled into secretory vesicles on the outside of the ER and trafficked to the PV where the PTEX translocon is located^14^,^15^.

In the context of KAHRP, it is clear that the sequence around PEXEL is not critical but the distance from the signal sequence is important for export, and this was directly linked to the efficiency of PEXEL processing. The steps in export after PMV are not yet clear; however, the Ac-xE/Q/D revealed by cleavage may be a recognition signal for receptors that link cargo inside the ER to coat proteins on forming vesicles outside the ER membrane^1^,^9^.

A previous study reported *P. falciparum* recombinant exported proteins bind PI(3)P with high affinity and the conserved PEXEL Arg, or Lys in PfEMP1, was responsible^24^. When we tested equivalent PEXEL and PfEMP1 proteins using the methods published by Bhattacharjee *et al.*^24^, we did not detect reproducible binding to PI(3)P. Indeed, consistent binding to PI(3)P by p40PX was dependent on the conditions used. Under such conditions, if binding for a recombinant exported protein was detected, it was neither PEXEL dependent nor PI(3)P specific. If the PEXEL did bind PI(3)P as a conserved mechanism for export in *P. falciparum*, it would be expected to occur for multiple proteins and irrespective of the signal peptide chosen or insertion of four unnatural alanine amino acids beyond the signal sequence; however, this was not observed. Interestingly, binding of GBP130 with either a native or mutant PEXEL to PI(3)P, PI(4)P and PI(5)P was seen using spot assays but our data indicate that this was mediated by a minor population of unfolded GBP130, as 1/20 amount of DnaK could block the interaction. Similar nonspecific binding to PI(3)P was reported for a *P. infestans* RxLR protein^28^. This raises the possibility that partially unfolded proteins may have contributed to the PI(3)P binding reported previously^24^. We conclude that some experimental conditions may permit some lipid binding but it is not PEXEL dependent, reproducible across all exported proteins or physiologically relevant.

PI(3)P is a product of PI3 kinase and is found on endosomal and lysosomal membranes in mammalian cells and yeast^36^,^49^. We and others^30^ have localized PI(3)P to the food vacuole and apicoplast membranes in *P. falciparum*. Bhattacharjee *et al.*^24^ reported this lipid is localized on the inner ER leaflet in a steady state for the first time in a eukaryote. However, there is no obvious gene that encodes an ER-resident PI(3) kinase in *P. falciparum* and PI(3)P recruitment to the ER in eukaryotes is rare, occurring only transiently during autophagy^50^. Autophagosome formation requires a protein complex but orthologues to some proteins appear to be absent from *Plasmodium* spp.^51^,^52^,^53^. Bhattacharjee *et al.*^24^ used fluorescent proteins fused to EEA1 and p40PX to infer PI(3)P is located in the ER. However, the FYVE finger of EEA1 is a poor PI(3)P sensor, because its binding to this lipid is very weak and occurs via strong co-binding to Rab5:GTP^36^,^54^,^55^. As Rab5:GTP is located on endosomal membranes, EEA1 provides little information about PI(3)P on non-endosomal membranes, and several publications have advised against using this protein for such purposes^36^,^55^. We used p40PX and Hrs, and their expression in *P. falciparum* showed accumulation at the membrane of lysosome-like food vacuoles and the apicoplast, suggesting PI(3)P is located at these membranes, consistent with its widely conserved role in endosomal trafficking. When p40PX-GFP was directed into the *P. falciparum* ER, it was not retained but secreted to the PV. In contrast, when Hrs-GFP was directed into the ER, it remained there but mutation of resides required for PI(3)P binding did not alter this localization. This suggests PI(3)P-independent retention of exogenous PI(3)P-binding biosensors can occur in the *P. falciparum* ER and may explain discordant results and conclusions of Bhattacharjee *et al.*^24^ compared with our work. These results highlight the need for caution when using such PI(3)P reporters in *P. falciparum*.

The PI(3)P hypothesis originated from research on oomycetes, where virulence effectors containing an RxLR motif are directed into plant host cells by binding to PI(3)P on the cell surface followed by endocytosis^39^,^56^, although this has now been challenged (reviewed in ref. 57). The mechanism for translocation of RxLR proteins into the plant host is unclear and is an area of debate. Although effector binding to PI(3)P on the plant cell surface has been described^39^,^56^, other studies have found that when PI(3)P binding is observed it occurs through regions other than the RxLR motif. In examples where the motif has been found to be important for effector entry, it is not via PI(3)P binding, because the RxLR motif does not bind phospholipids in these studies^28^,^58^,^59^,^60^,^61^.

The PEXEL of *Plasmodium* spp. has obvious similarities to the RxLR motif of oomycetes and it was therefore possible they share a common mechanism. Indeed, it was reported that RxLR directs export to *P. falciparum*-infected erythrocytes^24^,^25^, whereas insertion of the PEXEL into a *Phytophthora* protein permitted its endocytosis by plant cells^38^. We tested this for *P. falciparum*, in independent laboratories, by making identical as well as different constructs as those described^24^,^25^. None of the chimeric RxLR proteins from *P. infestans* and *P. sojae* were exported. These results are important, because a primary conclusion by Bhattacharjee *et al.*^24^ was that RxLR mediates export in *P. falciparum* in the absence of PMV cleavage^23^. In agreement with Bhattacharjee *et al.*^24^, we showed the RxLR motif is not a substrate for PMV cleavage and this provides a clear mechanistic explanation for why this sequence cannot mediate export in *P. falciparum*.

In conclusion, we find no reproducible evidence that PI(3)P binding in the ER is the mechanism for export of PEXEL proteins. Rather, the PEXEL is present at a conserved distance from the signal peptide that permits co-translational processing by PMV, to reveal a signal for the next step in export to the human erythrocyte^10^,^11^.

## Methods

### Plasmids for recombinant protein expression

*P. falciparum* exported proteins GBP130 (PF3D7_1016300), KAHRP (PF3D7_0202000), HRPII (PF3D7_0831800) and PfEMP1 (PF3D7_1240600) were expressed in this study. GBP130_66–196_ and GBP130_66–196_RLE>A were described previously^10^. Briefly, DNA encoding the proteins was codon optimized (Genscript) and cloned into pET11a using NdeI/BamHI (generating pGBP130_3Cmyc18His_ and pGBP130-3A_3Cmyc18His_). SacII followed by DNA encoding RGS18His was used in the constructs to improve Ni^2+^ binding, allowing stringent washing^62^. For the current study, GFP was incorporated into pGBP130_3Cmyc18His_ by PCR from pGlux.1 using oligos JB176/JB177, with JB177 encoding 6His, and cloned via SacII/BamHI, generating pGBP130_3CmycGFP6His_. GBP130 fragments 66–83, 66–88, 84–196 and 89–196 were amplified from pGBP130_3Cmyc18His_ using JB170/JB171, JB170/JB174, JB172/JB173 and JB175/JB173, respectively, and cloned into pGBP130_3CmycGFP6His_ by NdeI/SacII, excising 3Cmyc. DNA encoding KAHRP_35–72_, KAHRP_35–72_ RLQ>A, HRPII_22–65_ WT, HRPII_22–65_ RLE>A, PfEMP1_1–60_ and PfEMP1_1–60_ K>A (PFL1960w), was codon optimized (Genscript) and cloned into pGBP130_3CmycGFP6His_ by NdeI/SacII. pGEX-p40PX(aa1–148) was purchased from Addgene (19008), deposited by Michael Yaffe. See Supplementary Table 1 for more information.

### Recombinant protein expression and purification

*Escherichia coli* BL21.DE3 was grown in Luria broth with 100 μg ml^−1^ carbenicillin at 37 °C, 200 r.p.m., to optical density (600 nm) 0.5, induced with 1 mM isopropyl-1-thio-β-D-galactopyranoside (Astral), grown for 5 h at 30 °C, 200 r.p.m., collected by centrifugation (8,000*g*, 4 °C) and lysed in 50 mM NaH_2_PO_4_, 300 mM NaCl, 10 mM imidazole (Sigma-Aldrich) pH 8.0, containing 1 mg ml^−1^ lysozyme (Sigma), 1 mM phenylmethylsulfonyl fluoride (Sigma-Aldrich), 1 × Complete cocktail (Roche). DNAse (0.01 mg ml^−1^; Roche), RNAse (0.01 mg ml^−1^; Life Technologies) and MgCl_2_ (5 mM) was added on ice for 30 min. Debris was collected (11,000*g*) and supernatants filtered through 5.0- and 0.8-μM filters (Millipore), and proteins were purified using Ni-NTA sepharose (Roche) in 10 ml chromatography columns (Bio-Rad), washed in 50 mM NaH_2_PO_4_, 300 mM NaCl, 20 mM imidazole pH 8.0 and eluted in 100 mM followed by 200 mM imidazole. Elutions were viewed by SDS–PAGE and Coomassie blue staining. All GFP-tagged proteins were fluorescent. Fractions were pooled, dialysed against 150 mM NaCl, 20 mM HEPES pH 7.5, using Spectra/Por Dialysis tubing (MWCO 12–14,000; SpectrumLabs) at 4 °C overnight and concentrated to 2 mg ml^−1^ in Centricon YM-10 (MWCO 10,000) concentrators by centrifugation at 4,000*g* for 30 min at 4 °C. Size-exclusion chromatography was performed using Superdex 200 10/300 columns.

Recombinant p40PX(1–148) was expressed in *E. coli* using similar methodology, except the cells were expressed for 16 h at 18 °C post isopropyl-1-thio-β-D-galactopyranoside induction. p40PX was purified using glutathione sepaharose and eluted with 20 mM reduced glutathione, followed by size-exclusion chromatography using a Superdex 200 10/300 column. Recombinant GST-p40PX was also purchased (Echelon Biosciences; G-0302).

### Preparation of liposomes

Liposomes were prepared as described by Bhattacharjee *et al.*^24^. PI(3)P-containing liposomes: solutions of 1.95 μmol PC and 0.52 μmol PE in CHCl_3_ (Avanti Polar Lipids; 850457C and 850757C), and 0.13 μmol D-*myo*-phosphatidylinositol 3-phosphate di-C16 (PI(3)P-diC16; Echelon Biosciences; P-3016) dissolved in CHCl_3_:MeOH:H_2_0 (1:2:0.8) were mixed and dried under nitrogen to give 2.6 μmol total lipid at a ratio of 75:20:5 (PC:PE:PI(3)P-diC16). Control liposomes: solutions of 2.08 μmol PC and 0.52 μmol PE in CHCl_3_ were mixed and dried under nitrogen to give 2.6 μmol total lipid at a ratio of 80:20 (PC:PE). Lipid films were resuspended in 2.6 ml, 20 mM Tris Cl pH 7.4, containing 0.16 M KCl and incubated at 65 °C for 1 h with regular vortexing, to create a lipid emulsion. Four hundred aliquots were transferred to cryovials (Nunc) and were freeze–thawed three times in liquid nitrogen followed by 37 °C water bath and vortexing. Emulsions in glass tubes were sonicated in a Branson 2200 water bath for 30–60 min, ensuring the temperature remained below 40 °C. Liposomes were at stored at 4 °C and used within 5 days.

### Binding to liposomes by SPR

SPR experiments were performed as described by Bhattacharjee *et al.*^24^ and others^63^,^64^ using a Biacore 3000 at 25 °C. Liposomes were coated onto an L1 sensor chip by injecting 100–300 μl of a 0.1-mM total lipid solution at 5 μl min^−1^ using 150 mM NaCl, 50 mM HEPES pH 7.5 as buffer. Control liposomes were injected over lane 1 and PI(3)P liposomes over lane 2. The amount injected was varied so that the total amount bound (typical equilibrium response of 3,000–6,000 response units^24^) was approximately equal between lanes. Following immobilization, lanes were washed with 3 × 10-μl 50 mM NaOH at 100 μl min^−1^. Before proceeding, 100 μl of 0.1 mg ml^−1^ BSA was injected over both lanes, to determine the level of residual nonspecific binding to the chip surface.

Recombinant proteins were passed over the liposome-coated L1 chip at a flow rate of 30 μl min^−1^ in 150 mM NaCl, 50 mM HEPES pH 7.5 buffer for 3 min at concentrations between 62.5 and 2,000 nM, and allowed to dissociate for 3 min. Binding to control liposomes (lane 1) was subtracted to yield the final sensorgrams, as described^24^. A pulse of 50 mM NaOH was applied at the end of each run to dissociate any bound protein. The chip surface was regenerated periodically by injecting 40 μl of 20 mM CHAPS at a flow rate of 30 μl min^−1^ and then re-coating with liposomes as described above. Req values were plotted against protein concentration and *K*_d_ values determined by nonlinear least-squares analysis of the binding isotherm using concentrations that spanned the *K*_d_.

### Binding to liposomes by ultracentrifugation

Liposomes and recombinant proteins were subjected to ultracentrifugation at 75,000*g* for 20 min at 25 °C separately. Liposomes and soluble recombinant proteins were resuspended to a final concentration of 1 mM total lipids and 5 μM recombinant proteins in either PBS pH 7.5 or 150 mM NaCl, 50 mM HEPES pH 7.5 as described^24^,^64^. Reactions were incubated for 20 min at 25 °C before ultracentrifugation again. Supernatants were collected and mixed with reducing sample buffer, pellets were resuspended in 100 μl of reducing sample buffer, and equal proportions were resolved by SDS–PAGE and visualized with Coomassie blue staining. Bands were quantified using a GS-800 Calibrated Densitometer (Bio-Rad).

### Binding to PI(3)P-coated beads by pull down

PI(3)P-coated beads and control beads were purchased from Echelon Biosciences (P-B003a and P-B000) and protein binding was performed according to the manufacturer's instructions. Briefly, 1 μg protein was incubated in binding buffer (150 mM NaCl, 0.2% NP40, 10 mM HEPES pH7.4) with 50 μl beads in 100 μl total volumes for 1 h at 25 °C. Beads were collected in Micro Bio-Spin columns (Bio-Rad), washed with 5 × 1-ml binding buffer and bound proteins eluted in 25 μl reducing sample buffer for 15 min at 25 °C. Eluates were resolved by SDS–PAGE and detected by immunoblotting with anti-GST (p40PX) or anti-GFP (exported proteins) antibodies. Ten per cent of the unbound fraction volume was loaded to visualize the proteins.

### Binding to lipid by ITC

Titrations were performed with a MicroCal iTC200 at 20 °C using proteins dialysed against 150 mM NaCl, 20 mM HEPES pH 7.5 as baits (200 μl) at 109 μM for HRPII WT, 58 μM for HRPII RLE>A, 49 μM for GBP130_66–88_ and 140 μM for Htp1 from *S. parasitica*^27^. Inositol 1,3 bisphosphate titrant was prepared at 2.4 mM and Fmoc-Tyr(SO_3_)-OH titrant at 5.12 mM. Titrations used 0.4 μl followed by 18 × 2 μl titration steps. The ITC stirring speed was 1,000 r.p.m. and the feedback gain mode was set to high.

### Binding to lipid spot membranes

Lipid spot membranes (Echelon Biosciences, S-6000, P-6001 and P6002) equilibrated in PBS containing 0.1% Tween 20 and 5% lipid-free BSA (PBS/Tween/BSA) were incubated with 20 μM recombinant proteins for 20 min at 25 °C. Membranes were washed thrice in PBS/Tween/BSA and bound proteins detected with mouse anti-Penta-His antibodies (1:10,000; Qiagen) followed by horseradish peroxidase-conjugated secondary antibodies (1:2,000; Silenius) and visualized by enhanced chemiluminescence (Amersham).

### Plasmids for transgenic parasites

*P. falciparum* expressing PfEMP3_1–82_ and KAHRP_1–96_ were generated previously^10^, as were parasites expressing KAHRP_1–70_ (ref. 65). DNA encoding PfEMP3 L>A, PfEMP3 4A RLQ>A, HRPIIs-AVR3a, HRPIIs-PH001D5, HRPII 4A, HRPII 4A RLE>A, HRPII 4A RLE>A Down, KAHRP Δ38–48, KAHRP Δ38–53, KAHRP Δ38–53 RLQ>A, KAHRP16Ala, KAHRP 10Ala and KAHRP 5Ala was synthesized by Epoch Biolabs or Genscript, and cloned in-frame with GFP in pGlux.1 using XhoI/XmaI. p40PX-GFP: DNA encoding the PX domain from human p40phox was amplified from p40PX-EGFP (Addgene 19010) using primers p40_A_F/p40_K_R and cloned into pARL2-GFP^66^ using AvrII/KpnI (p40-GFP). STEVORss-p40PX: DNA encoding the first 30 amino acids (signal sequence) of STEVOR was released from pARL-STEVOR^(1–30)^ (ref. 66) with XhoI and AvrII, and cloned into p40-GFP (pSTEV-40-GFP). STEVORss-p40PX-GFP_SDEL_: DNA encoding GFP-SDEL was amplified from pARL2-GFP using GFP_K_F/GFP_SDEL_X_R and cloned into pSTEV-40-GFP using KpnI/XmaI (pSTEV-40-GFP^SDEL^). ACPss-p40PX: human p40PX (residues 1–148) was PCR amplified from human complementary DNA using oligo 5′ACPsp-PX40p, which also encodes the first 20 amino acids (signal sequence) of ACP, and oligo 3′KpnI-PX40-rev (blunt-KpnI). The amplicon was cloned into pGFP_glmS^67^ digested with BglI, blunted using Klenow and re-digested with KpnI. Hrs-GFP: DNA encoding 2xFYVE from mouse Hrs separated by QGQGS was amplified from pGFP-2xFYVE^30^ using primers HRS_FYVE_AvrII_F/HRS_FYVE_KpnI_R and cloned into pARL2-GFP using AvrII/KpnI (pHRS-GFP). STEVORss-HRS: DNA encoding the first 30 amino acids (signal sequence) of STEVOR was released from pARL-STEVOR^(1–30)^ with XhoI and AvrII, and cloned into pHRS-GFP (pSTEV-HRS-GFP). STEVORss-HRS-GFP^SDEL^: DNA encoding STEVORss-Hrs was released from pSTEV-HRS-GFP using AvrII/KpnI and cloned into similarly restricted pSTEV-40-GFP^SDEL^. STEVORss-HRS_C2158_-GFP: a fragment encoding 2xFYVE^MUT^ (containing the double C^215^S mutation) was amplified from p-ddFYVEm^30^ using primers HRS_FYVE_AvrII_F and HRS_FYVE_KpnI_R, and cloned into pSTEV-HRS-GFP using AvrII/KpnI (pSTEV-HRS^mut^GFP).

To make plasmids encoding REX3s-Avh5 and REX3ss-Avr1b, DNA fragments Rex3(1–43)-Avr1b(22–71) and Rex3(1–43)-Avh5(20–63) were PCR amplified from truncated REX3-GFP^68^ using primers REX3fwK^22^, and reverse primers Rex3(1–43)-Avr1b(22–71)-KpnI-rev and Rex3Avh5(20–63)-AvrII-rev, respectively. These inserts were cloned KpnI/AvrII into pARL1GFP^69^. See Supplementary Table 1 for more information.

### Oligonucleotides

Oligonucleotide sequences are shown in Supplementary Table 2. Oligonucleotides were made by Geneworks, Integrated DNA Technologies, Life Technologies or Sigma.

### Parasites

*P. falciparum* 3D7 parasites were used for this study as was the case for Bhattacharjee *et al.*^24^ Parasites were transfected and grown in O^+^ human erythrocytes with selection under 2–5 nM WR99210 (a gift from Jacobus Pharmaceuticals), as described^70^.

For MS, 900 ml of trophozoite-infected erythrocytes grown to 10% parasitaemia in 5 nM WR99210 were purified by Varia Macs magnet separation through D columns (Miltenyi Biotech), fractionated with 0.15% saponin containing 1 × Complete protease inhibitors (Roche) and stored at −80 °C.

### Microscopy and quantification of export and statistics

For immunofluorescence microscopy, cells were fixed in 4% paraformaldehyde, 0.01% glutaraldehyde (ProSciTech) for 30 min, to preserve GFP fluorescence, and permeabilized in 0.1% Triton X-100 in PBS for 10 min. Cells were probed with rabbit anti-CRT (1:600, MR4, MRA-308), rabbit anti-ACP (1:600, a kind gift from Geoff McFadden), rabbit anti-ERC (1:500, a kind gift from Leanne Tilley), rabbit anti-EXP2 (1:200, a kind gift from Jana McBride), rabbit anti-PMV (1:750)^43^, or mouse anti-PMV (1:200, MR4, MRA-815A, contributed by Dan Goldberg) diluted in PBS containing 3% BSA for 1 h followed by Alex Fluor 594-conjugated secondary immunoglobulin G antibodies (1:1,000; ThermoFisher Scientific) (catalogue number A-11032 and A-11037) for 1 h and nuclei were labelled with 0.2 μg ml^−1^ 4′-6-diamidino-2-phenylindole (Roche) diluted in Vectorshield (Vector Labs). Samples were imaged on several platforms as follows: (1) a Deltavision Elite microscope with micrographs collected on a Coolsnap HQ2 charge-coupled device camera through a Olympus × 100 UPlanSApo numerical aperture 1.4 objective with SoftWorx software; (2) a Zeiss Axio Scope M1 microscope equipped with a × 100/numerical aperture 1.4 oil-immersion lens using a Hamamatsu Orca C4742–95 camera and Zeiss AxioVision software; and (3) a Zeiss Cell Observer epifluorescence imaging system. Images were assembled with ImageJ Fiji (versions 1.42q and 1.47d), Adobe Photoshop and Illustrator CS5 or CS6, or Corel PHOTO-PAINT X4.

For quantification of exported GFP, the number of infected erythrocytes with exported protein, or with protein localized in the ER/PV/mix of both, was counted using 20 cells per construct represented as a percentage. Live GFP images were blinded and the localization determined by two independent observers. For quantification of exported GFP in Fig. 5, *Z*-stacks of live-GFP images of synchronous parasites were captured on a Deltavision Elite microscope using a × 100 objective. At least 20 cells per condition were imaged using the same exposure settings, to allow quantitative analysis of GFP intensity in the host cell (signal outside the parasite and PV) between chimeras. All statistical analyses were performed using Prism 6 and mean differences were compared using the Mann–Whitney *t*-test.

### Peptide cleavage and RP-HPLC

PMV-agarose was prepared by mixing goat anti-HA agarose (Sapphire Bioscience) with parasite lysates containing PMV-HA^10^. For peptide cleavage assays, 2 nmol synthetic peptides (Genscript, LifeTein) were mixed with 5 μl PMV-agarose (or PBS control) in 25 mM Tris HCl, 25 mM MOPS pH 6.4 and incubated at 37 °C for 24 h. Reactions were passed through Micro Bio-Spin columns (Bio-Rad), to remove agarose, and analysed with an Agilent 1100 modular HPLC^10^ and MS (liquid chromatography–tandem MS (LC–MS/MS), see below).

### Subcellular fractionation and immunoblotting

Parasite-infected erythrocytes were magnet purified on CS columns (Miltenyi Biotech) and solubilized in 4 × Laemmli buffer as whole infected cells, or permeabilized in 100 units per ml tetanolysin (Sigma) or 0.15% saponin containing 1 × Complete protease inhibitors as described previously^9^, before pellet and supernatant fractions were resuspended in 4 × Laemmli buffer. Samples were boiled, separated by SDS–PAGE and transferred to nitrocellulose membrane. Membranes were blocked in PBS-T containing 10% skim milk and proteins detected using mouse anti-GFP (Roche; 1:1,000, catalogue number 11814460001), rabbit anti-Aldolase (1:1,000)^46^, rabbit anti-PfHSP70 (1:4,000)^46^, mouse anti-Penta-His (1:4,000, Qiagen, catalogue number 34460) or rabbit anti-GST (1:1,000; WEHI Antibody Facility) antibodies followed by horseradish peroxidase-conjugated secondary antibodies (1:2,000; Cell Signalling Technology, catalogue numbers 7074, 7076 and 7077) and visualized by enhanced chemiluminescence (Amersham). Images were scanned using a GS-800 Calibrated Densitometer (Bio-Rad) at 400 dpi using Quantity One v4.6.9 software (Bio-Rad). Uncleaved, signal peptide-cleaved and PEXEL-cleaved proteins were deduced based on predicted molecular weight or by MS^9^,^10^,^11^,^43^. Full-length versions of all gels and blots are provided in Supplementary Information.

### Protein labelling IPs and densitometry

Synchronous early trophozoites were magnet purified on CS columns (Miltenyi Biotech) and incubated in Met/Cys-free medium for 30 min at 37 °C before addition of 300 μCi ml^−1 35^S-Met/Cys (Perkin/Elmer) to the medium. Parasites were incubated at 37 °C, pelleted and snap frozen in ethanol/dry ice bath at the indicated times and stored at −80 °C. Samples were solubilized in 25 mM Tris-HCl pH 8.0, 150 mM NaCl, 2 mM EDTA, 1% Triton X-100 and Complete inhibitor cocktail (Roche), and GFP species were immunopurified with α-GFP agarose (MBL; catalogue number D153-8) at 4 °C for 1 h. Proteins were resolved by SDS–PAGE, visualized by autoradiography and quantified with a GS-800 Calibrated Densitometer (Bio-Rad).

### Lopinavir experiment

Synchronous early trophozoites were magnet purified on CS columns (Miltenyi Biotech) and incubated with dimethyl sulfoxide, 50 μM lopinavir, 150 ng ml^−1^ chloroquine or 100 ng ml^−1^ artemisinin for 7 h before parasite proteins were radiolabelled as described above, but using 800 μCi ml^−1 35^S-Met/Cys for 15 min. Pellets were saponin treated (0.15%), snap frozen and stored at −80 °C. Samples were solubilized in Laemmli buffer, resolved by SDS–PAGE, visualized by autoradiography and quantified as above.

### PI(3)P ELISA assays

ELISA assays were performed using a PI(3)P Mass ELISA kit (Echelon Biosciences, K-2500s) according to manufacturer's instructions and Bhattacharjee *et al.*^24^. Synchronous early trophozoites at 10% parasitaemia were magnet purified on CS columns (Miltenyi Biotech) and solubilized in lipid-binding buffer (20 mM Tris HCl pH 7.5, 150 mM NaCl, 0.25% Igepal, 1 mM EDTA) with Complete inhibitor cocktail (Roche) by rotating at 4 °C for 1 h. Insoluble debris was removed by centrifugation (20,800*g*) for 10 min at 4 °C. GFP species were immunopurified as above, beads were washed with lipid-binding buffer and proteins eluted in 100 mM Glycine pH 2.6, 500 mM NaCl. Elutions were immediately neutralized in 1 M Tris HCl pH 8.0. A total of five IPs was performed per sample. As conducted previously^24^, proteins were quantified by immunoblotting using mouse α-GFP (Roche; 1:1,000, catalogue number 11814460001) followed by densitometry of different exposures within the linear range. Equal protein concentrations were obtained by dilution and PI(3)P was then extracted from each protein and detected using the mass ELISA kit. Absorbance was read at 450 nm on an Envision fluorescence plate reader (Perkin-Elmer).

### Mass spectrometry

HRPII-PH001D5 protein bands were in-gel digested with AspN (Promega) and eluted peptides, or synthetic peptides incubated with PMV-agarose, were fractionated by reversed-phase liquid chromatography on a nanoflow HPLC system (1200 series, Agilent, USA) using a nanoAcquity C18 150 mm × 0.15 mm I.D. column (Waters, USA) developed with a linear 60- min gradient with a flow rate of 0.5 μl min^−1^ at 45 °C from 100% solvent A (0.1% formic acid in Milli-Q water) to 60% solvent B (0.1% formic acid, 60% acetonitrile and 40% Milli-Q water). The nano HPLC was coupled on-line to an LTQ-Orbitrap mass spectrometer equipped with a nanoelectrospray ion source (ThermoFisher Scientific) for automated MS/MS. Up to five most intense ions per cycle were fragmented and analysed in the linear trap, with target ions already selected for MS/MS being dynamically excluded for 3 min.

### Mass spectra database searching

LC–MS/MS data were searched against a custom decoy database comprising *Plasmodium* sequences from the LudwigNR non-redundant protein database (version Q211, 249275 entries), common contaminants (239 entries) and chimeric *P. falciparum* PEXEL constructs (44 entries), as well as their reverse sequences as described previously^9^. Peak lists for each nano LC–MS/MS run were used to search MASCOT v2.2.04 search algorithm (Matrix Science, UK) provided by the Australian Proteomics Computational Facility ( www.apcf.edu.au). Scaffold_3.6.2 (Proteome Software Inc.) was employed to validate MS/MS-based peptide and protein identifications. Peptide identifications were accepted if they could be established at >95.0% probability as specified by the Peptide Prophet algorithm. Extracted ion chromatograms of manually validated peptide sequences were generated using Excalibur v2.0 (ThermoFisher Scientific).

## Additional information

**How to cite this article:** Boddey, J. A. *et al.* Export of malaria proteins requires co-translational processing of the PEXEL motif independent of phosphatidylinositol-3-phosphate binding. *Nat. Commun.* 7:10470 doi: 10.1038/ncomms10470 (2016).

## Supplementary Material

Supplementary Information Supplementary Figures 1-11 and Supplementary Tables 1-2 and Supplementary References
