# Supplementary material for: Export of malaria proteins requires co-translational processing of the PEXEL motif independent of phosphatidylinositol-3-phosphate binding
Source: Nat Commun. 2016 Feb 1;7:10470. doi: 10.1038/ncomms10470 (PMC4740378; doi:10.1038/ncomms10470)
Supplement: Supplementary Information — Supplementary Figures 1-11 and Supplementary Tables 1-2 and Supplementary References [file ncomms10470-s1.pdf]

Supplementary information

Supplementary Figures

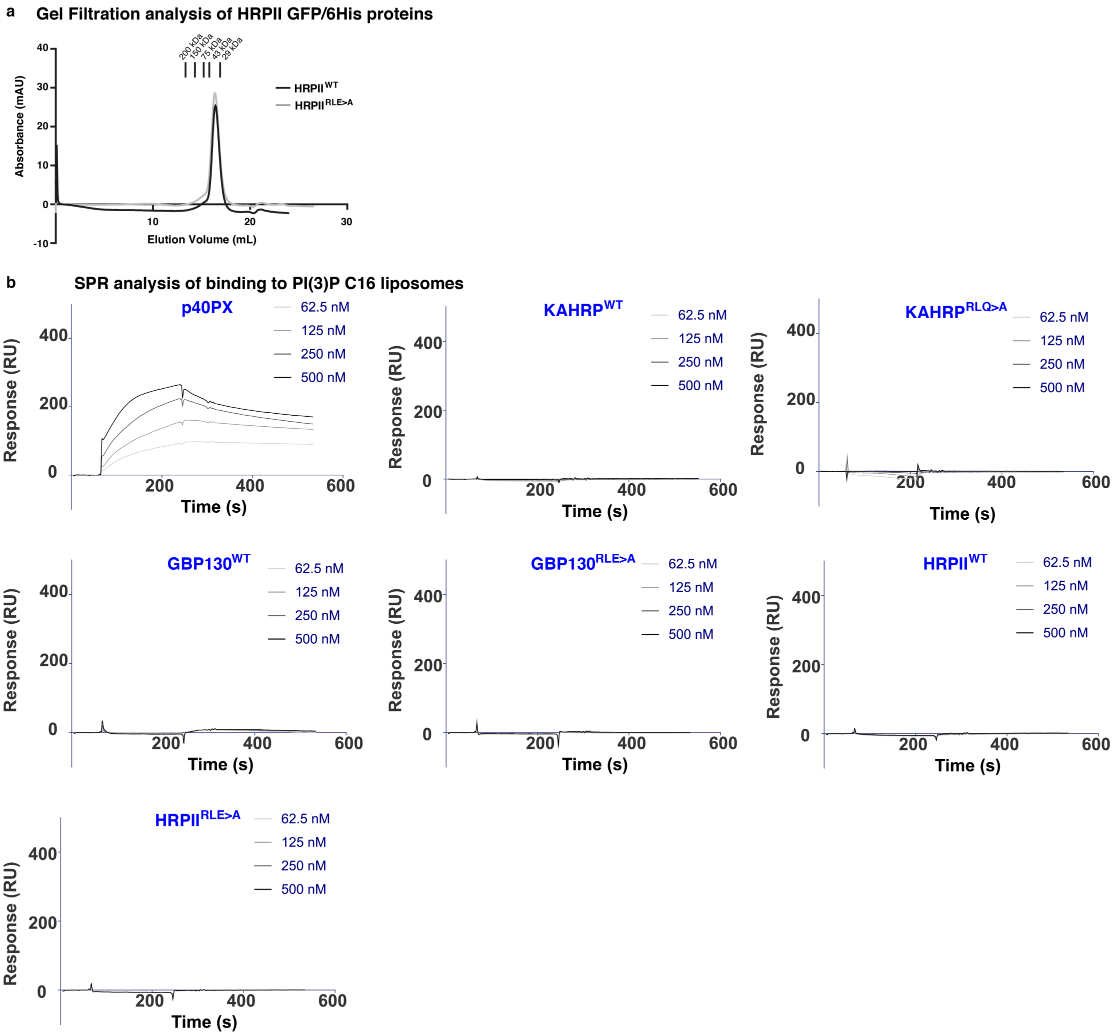

Supplementary Figure 1.

Recombinant PEXEL proteins do not bind PI(3)P. (a) Gel filtration analysis of HRPII^{WT} and HRPII$^{RLE>A}$ proteins shows the proteins are monomeric. **(b)** Surface plasmon resonance (SPR) analysis of recombinant protein binding to a PI(3)P liposome-coated chip. Binding to PC/PE control liposomes was subtracted to give the presented sensorgrams as performed in [1]. Recombinant p40PX (PI(3)P Grip from Echelon Biosciences) bound the liposomes (Kd 196 μM). KAHRP, GBP130 and HRPII proteins with a native PEXEL (WT) do not bind PI(3)P liposomes more than proteins possessing the corresponding mutant PEXEL (RLQ>A and RLE>A, respectively). Experiments were performed in triplicate.

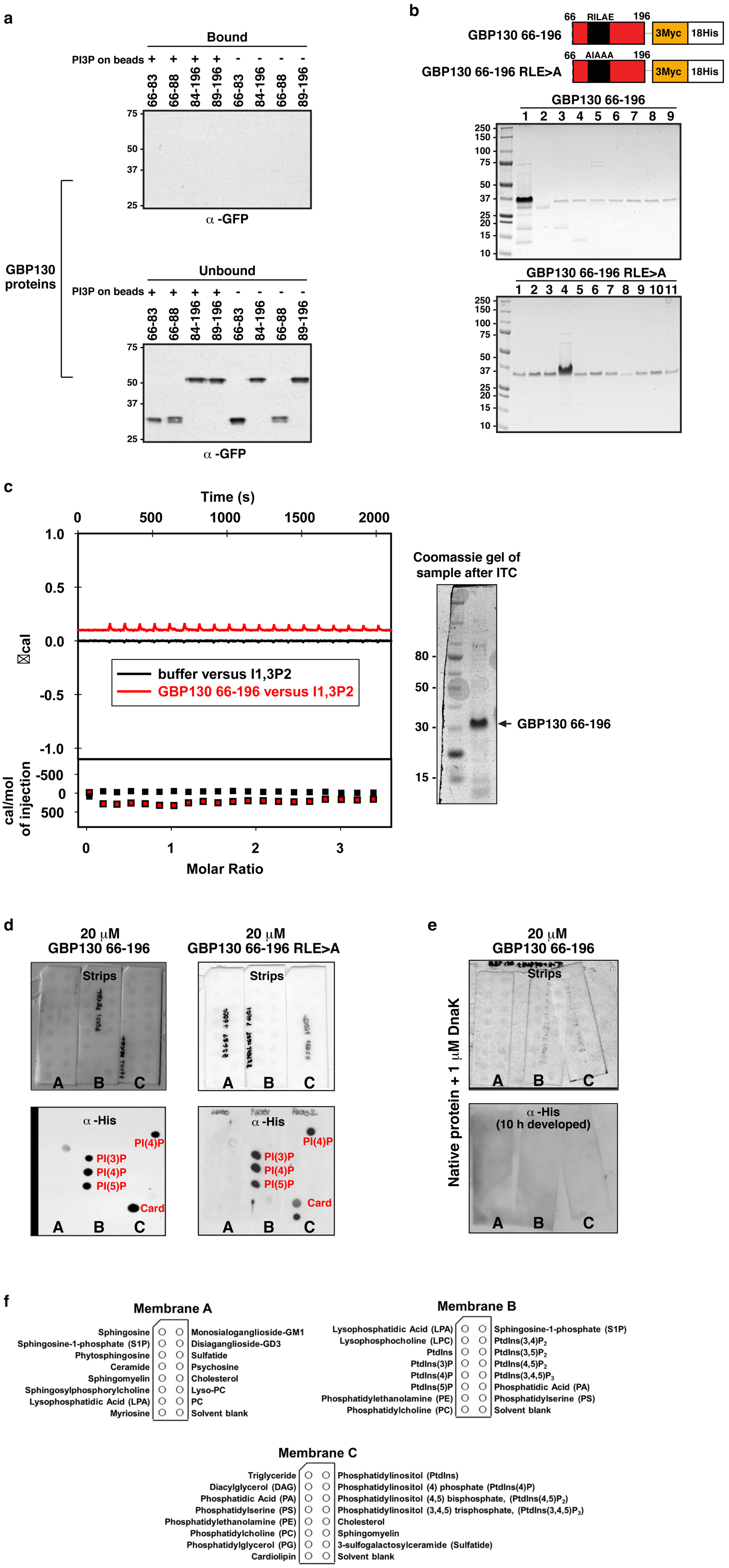
a
Bound
PI3P on beads + + + + - - - -
66-83 66-88 84-196 89-196 66-83 84-196 66-88 89-196
75 50 37 25
α -GFP
GBP130 proteins
Unbound
PI3P on beads + + + + - - - -
66-83 66-88 84-196 89-196 66-83 84-196 66-88 89-196
75 50 37 25
α -GFP
b
66 RILAE 196
GBP130 66-196
3Myc 18His
66 AIAAA 196
GBP130 66-196 RLE>A
3Myc 18His
GBP130 66-196
1 2 3 4 5 6 7 8 9
250 150 100 75 50 37 25 20 15 10
GBP130 66-196 RLE>A
1 2 3 4 5 6 7 8 9 10 11
250 150 100 75 50 37 25 20 15 10
c
Time (s)
0 500 1000 1500 2000
1.0 0.5 0.0 -0.5 -1.0
cal
buffer versus I1,3P2
GBP130 66-196 versus I1,3P2
-500 0 500
cal/mol of injection
0 1 2 3
Molar Ratio
Coomassie gel of sample after ITC
80 50 30 15
GBP130 66-196
d
20 μM GBP130 66-196
Strips
A B C
α -His
PI(4)P
PI(3)P
PI(4)P
PI(5)P
Card
A B C
20 μM GBP130 66-196 RLE>A
Strips
A B C
α -His
PI(4)P
PI(3)P
PI(4)P
PI(5)P
Card
A B C
e
20 μM GBP130 66-196
Native protein + 1 μM DnaK
Strips
A B C
α -His (10 h developed)
A B C
f
Membrane A
Sphingosine | Monosialoganglioside-GM1
Sphingosine-1-phosphate (S1P) | Disiaganglioside-GD3
Phytosphingosine | Sulfatide
Ceramide | Psychosine
Sphingomyelin | Cholesterol
Sphingosylphosphorylcholine | Lyso-PC
Lysophosphatidic Acid (LPA) | PC
Myriosine | Solvent blank
Membrane B
Lysophosphatidic Acid (LPA) | Sphingosine-1-phosphate (S1P)
Lysophosphocholine (LPC) | PtdIns(3,4)P2
PtdIns | PtdIns(3,5)P2
PtdIns(3)P | PtdIns(4,5)P2
PtdIns(4)P | PtdIns(3,4,5)P3
PtdIns(5)P | Phosphatidic Acid (PA)
Phosphatidylethanolamine (PE) | Phosphatidylserine (PS)
Phosphatidylcholine (PC) | Solvent blank
Membrane C
Triglyceride | Phosphatidylinositol (PtdIns)
Diacylglycerol (DAG) | Phosphatidylinositol (4) phosphate (PtdIns(4)P)
Phosphatidic Acid (PA) | Phosphatidylinositol (4,5) bisphosphate, (PtdIns(4,5)P2)
Phosphatidylserine (PS) | Phosphatidylinositol (3,4,5) trisphosphate, (PtdIns(3,4,5)P3)
Phosphatidylethanolamine (PE) | Cholesterol
Phosphatidylcholine (PC) | Sphingomyelin
Phosphatidylglycerol (PG) | 3-sulfogalactosylceramide (Sulfatide)
Cardiolipin | Solvent blank

Supplementary Figure 2. Assessment of PI(3)P binding by GBP130 proteins. (a) Immunoblot with anti-GFP antibodies shows that recombinant GBP130 protein fragments 66-83, 66-88, 84-196 and 89-196 (see Figure 1a) do not bind to PI(3)P-coated beads (+) or control beads (-) that were eluted with SDS-PAGE sample buffer. 10% of the unbound fraction volume was probed by immunoblot to visualize the proteins. The experiment was conducted at the same time as Figure 1d, where p40PX bound the PI(3)P-coated beads. Experiments were performed in triplicate. **(b)** Schematic of recombinant GBP130 66-196 proteins generated previously [2]. Residues 66-196 of GBP130, containing the PEXEL motif, or the PEXEL mutation RLE>A, were fused to 3cMyc epitopes and 18His residues. Coomassie blue-stained gels showing serial elutions of GBP130 proteins following expression in *E. coli* and absorptive chromatography. The last four fractions of each were used in subsequent experiments. **(c)** Isothermal calorimetry (ITC) of GBP130 66-196 (85 μM) in solution with the head group of PI(3)P, inositol 1,3 bisphosphate (I1,3P2), show no evidence of binding. The titration was similar between I1,3P2 (1.25 mM) into dialysis buffer (150 mM NaCl, 20 mM HEPES, pH 7.4) (negative control) and the same buffer containing GBP130 66-196. After ITC, the GBP130 66-196 protein sample was shown to be present by SDS-PAGE and Coomassie blue stain (right, arrow). **(d)** Binding of GBP130 66-196 and GBP130 66-196 RLE>A proteins (20 μM) to PI(3)P, PI(4)P, PI(5)P (membrane B), and PI(4)P and cardiolipin (card) (membrane C) on lipid spot membranes. Binding of both proteins shows it is not PEXEL-specific. **(e)** Binding of GBP130 66-196 (20 μM) to lipid spot membranes is blocked by 1 μM of the *E. coli* chaperone DnaK, which represents 5% of the total GBP130 amount added. Membranes were developed for 10 hours. **(f)** The identity of the lipids in each spot on membranes A, B and C are shown.

a

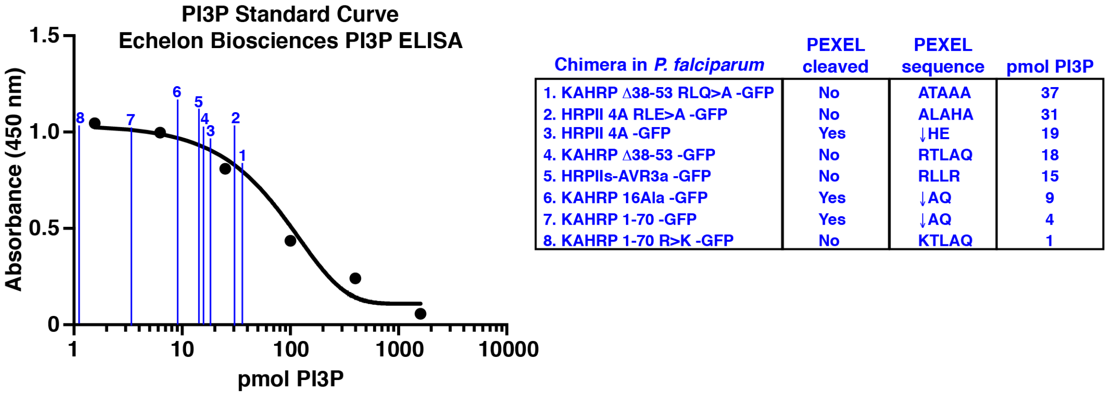

Chimera in *P. falciparum*	PEXEL cleaved	PEXEL sequence	pmol PI3P
1. KAHRP Δ38-53 RLQ>A -GFP	No	ATAAA	37
2. HRPII 4A RLE>A -GFP	No	ALAHA	31
3. HRPII 4A -GFP	Yes	↓HE	19
4. KAHRP Δ38-53 -GFP	No	RTLAQ	18
5. HRPIIs-AVR3a -GFP	No	RLLR	15
6. KAHRP 16Ala -GFP	Yes	↓AQ	9
7. KAHRP 1-70 -GFP	Yes	↓AQ	4
8. KAHRP 1-70 R>K -GFP	No	KTLAQ	1

b

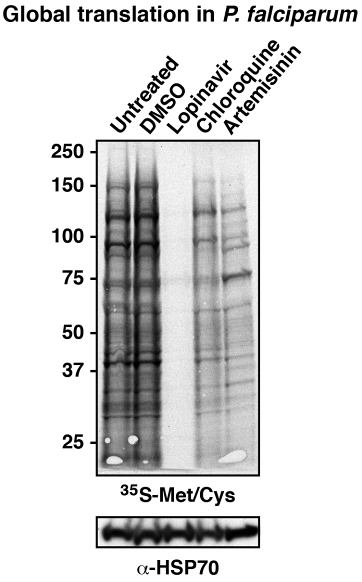

Supplementary Figure 3. PEXEL and RxLR chimeras do not bind PI(3)P in *P. falciparum*. (a) Standard curve generated using the PI(3)P standards provided in the Echelon Biosciences PI(3)P Mass ELISA kit. Blue lines indicate the pmol of PI(3)P bound to each chimera immunoprecipitated from *P. falciparum* and the deduced concentrations are listed in the Table in descending order (right). Bound PI(3)P was determined by measuring the optical density at 450 nm in biological duplicates. Data shown is one of two repeated experiments showing similar results. Whether the PEXEL in each chimera is cleaved by plasmepsin V, and the remaining PEXEL sequence thereafter, is indicated in the table. **(b).** Assessment of global protein synthesis in *P. falciparum*-infected erythrocytes following treatment with DMSO, lopinavir (50 μM), chloroquine (150 ng/ml) or artemisinin (100 ng/ml) for 7 hr prior to addition of radio-labelled amino acids to the culture medium for 15 min. DMSO had no effect compared to the untreated control; however, lopinavir treatment almost completely blocked translation. The positive control treatments chloroquine and artemisinin also had

negative effects on translation, likely because the parasites were dying from the 7 hr treatment. HSP70 levels were probed by immunoblot following radiolabelling.

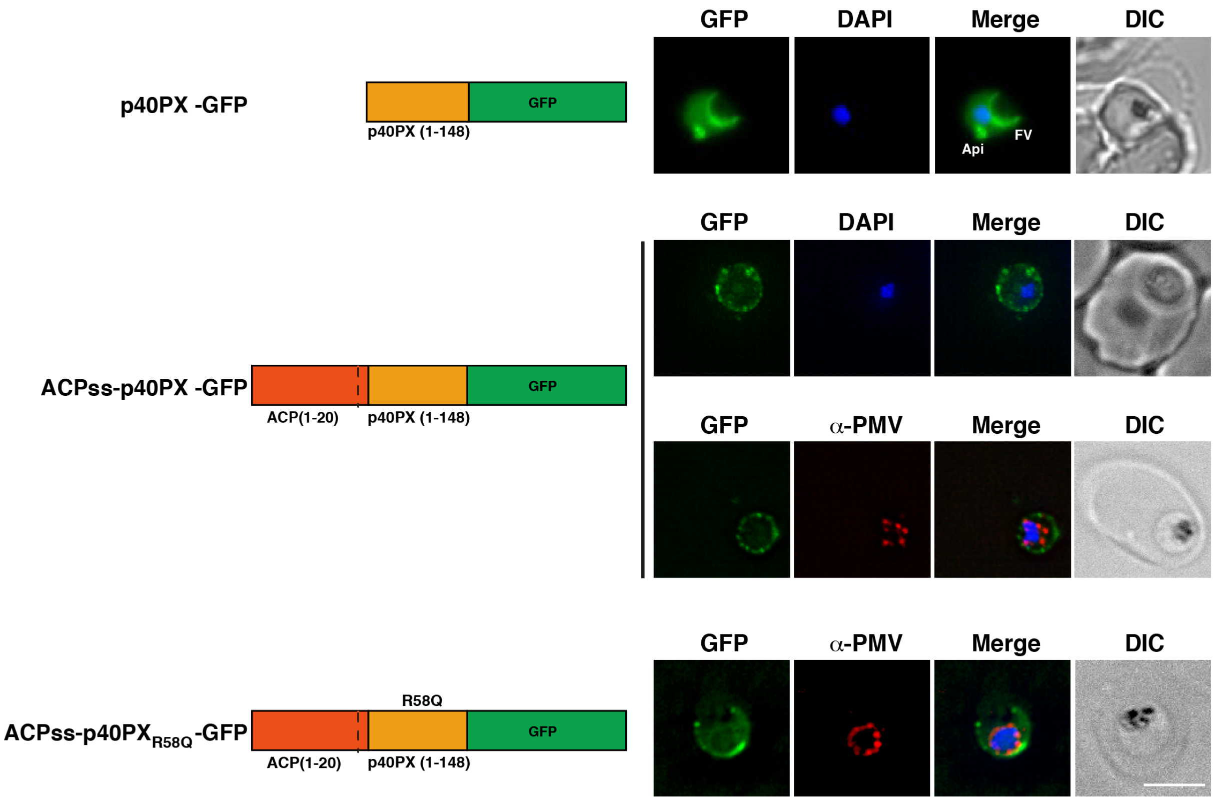

Supplementary Figure 4. Expression of p40PX in *P. falciparum* using the ACP signal sequence. Expression of p40PX-GFP in *P. falciparum* localizes the protein to the food vacuole membrane (FV) and apicoplast (Api). Middle: Fusion of p40PX-GFP to the signal peptide from acyl carrier protein (ACP) results in entry into the secretory pathway and localization at the parasitophorous vacuole in *P. falciparum*-infected erythrocytes. The PI(3)P reporter was not observed in the parasite ER, which was labelled with anti-PMV (plasmepsin V) antibodies. Mutation of the PI(3)P binding residue R58Q in p40PX (ACPss-p40PX$_{R58Q}$-GFP) results in localization at the parasitophorous vacuole, similar to the un-mutated form, and faint ER staining. Scale bar is 5 μM.

Figure S5

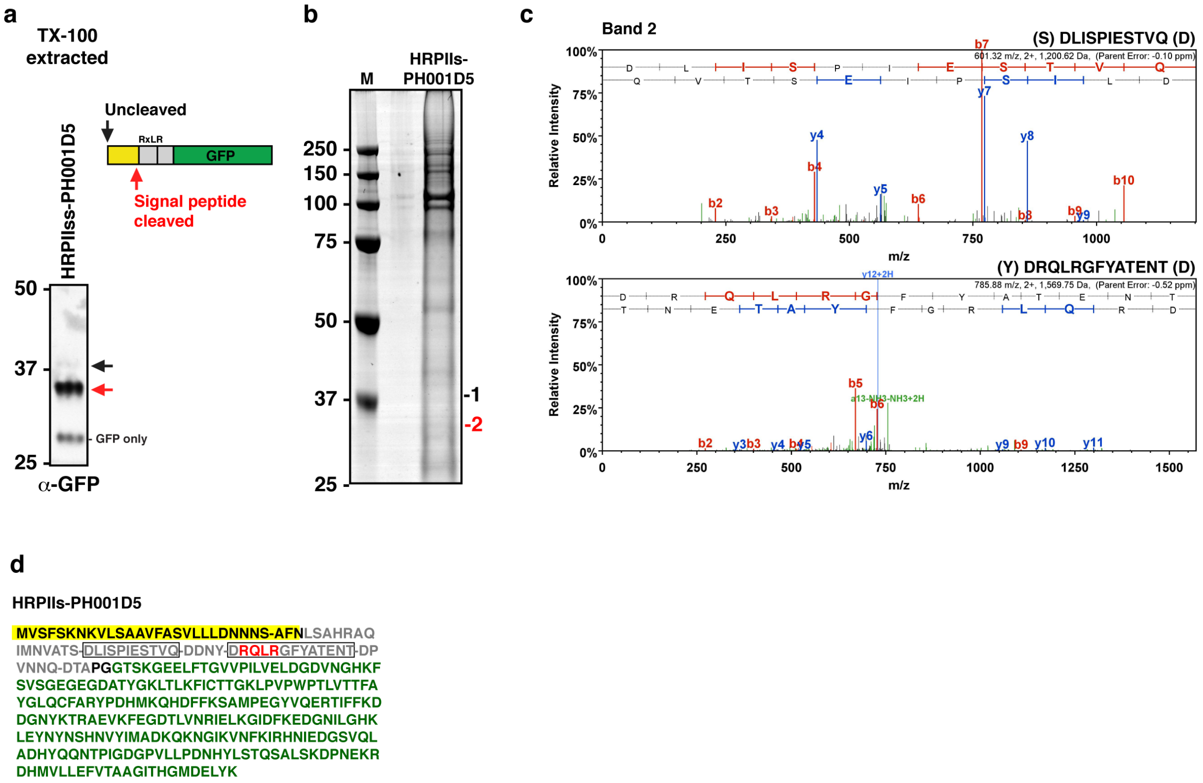

Supplementary Figure 5. The RxLR motif is not cleaved by plasmepsin V in *P. falciparum*. (a) Immunoblot with anti-GFP antibodies of HRPIIss-PH001D5 following affinity purification from TX-100 extracted transgenic *P. falciparum* lysates. The major species is signal peptide cleaved (32.8 kDa; red arrow), while uncleaved (35.8 kDa; black arrow) and 'GFP only' from the food vacuole are also present. The uncleaved species may be refractory to TX-100 extraction. **(b)** Coomassie blue stained gel of HRPIIss-PH001D5 proteins purified from *P. falciparum* TX-100 lysates by affinity chromatography with anti-GFP agarose. Bands 1 and 2 (labeled on right) were excised from the gel, digested with AspN (Promega) and subjected to LC-MS/MS for peptide identification. **(c)** Mass spectra of peptides detected from band 2 in panel (B) (the signal peptide cleaved species) that are either N-terminal to the RxLR motif (DLISPIESTVQ) but still C-terminal of the signal sequence (upper spectrum), or which contain the RxLR motif intact (DRQLRGFYATENT) showing it was not cleaved by plasmepsin V in *P. falciparum*-infected erythrocytes (lower spectrum) are shown. **(d)** Amino acid sequence of HRPIIss-PH001D5, showing the signal sequence (yellow) and signalP prediction (dash in the yellow region), the Oomycete effector sequence (grey) with RxLR sequence shown in red, the predicted AspN cleavage sites (dashes in the grey region) and GFP (green). The peptides detected by LC-MS/MS are boxed.

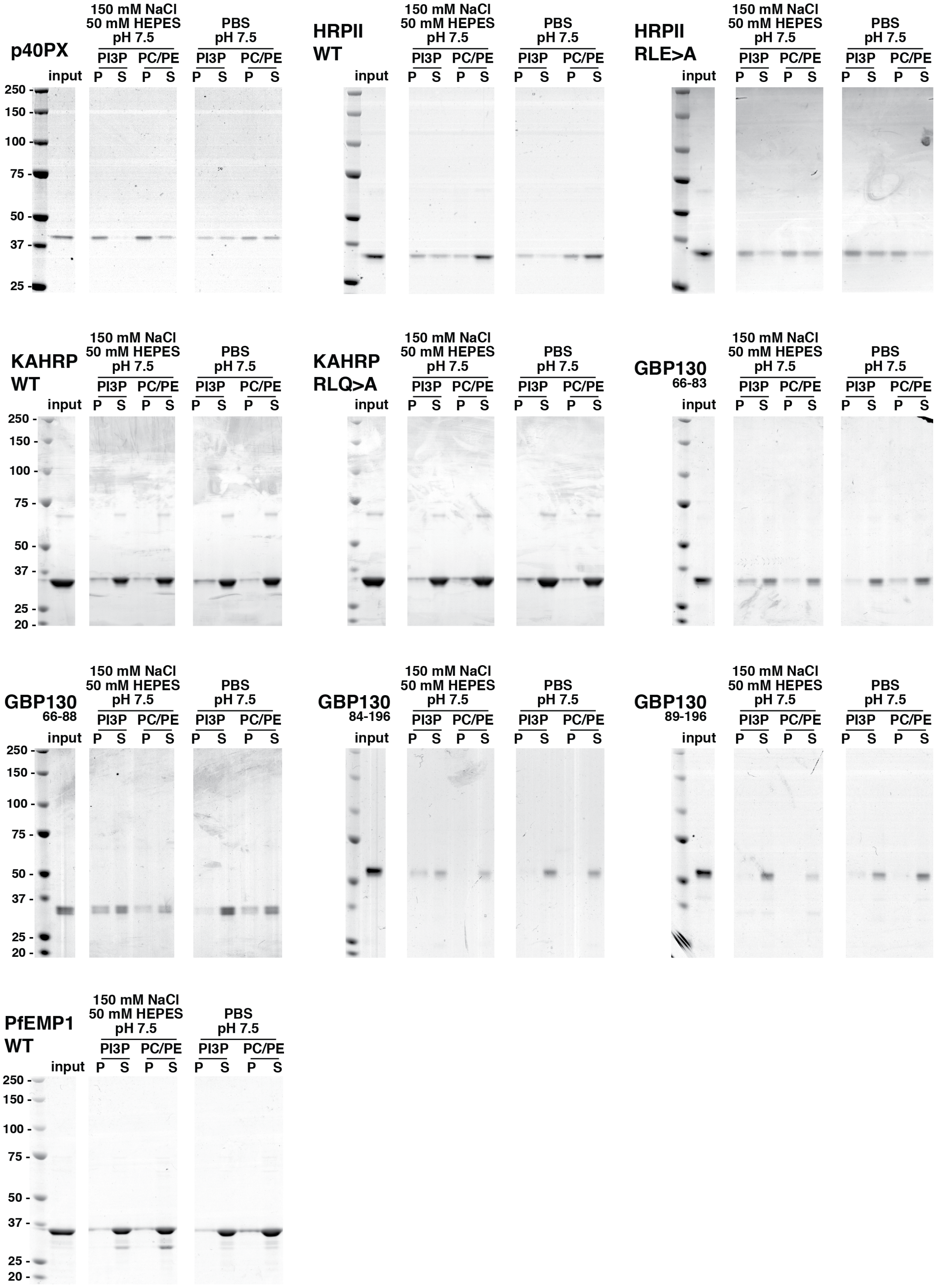

Supplementary Figure 6. Binding of proteins to PI(3)P liposomes or control PC/PE liposomes, determined by ultracentrifugation. Full-length gels from Fig. 1d. Pellet and

supernatant fractions were resolved by SDS-PAGE and Coomassie blue stain. Proteins were ultracentrifuged in buffer to remove potential aggregates prior to incubation with liposomes, explaining why input and sum of pellet and supernatant sometimes differ. Experiments were performed in triplicate.

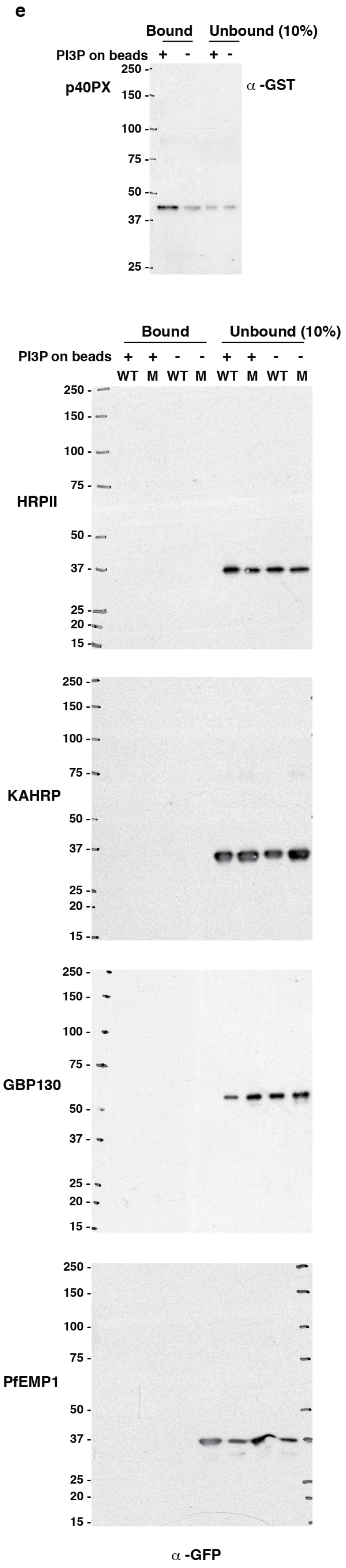
e
Bound
Unbound (10%)
PI3P on beads + - + -
p40PX
250
150
100
75
50
37
25
α -GST
Bound
Unbound (10%)
PI3P on beads + + - - + + - -
WT M WT M WT M WT M
HRPII
KAHRP
GBP130
PfEMP1
250
150
100
75
50
37
25
20
15
α -GFP

Supplementary Figure 7. Binding of p40PX and *P. falciparum* proteins with native PEXEL (WT) or mutant PEXEL (M) to PI(3)P-coated beads (+) or control beads lacking lipid (-). Full-length blots from Fig. 1e. Bound protein was eluted in SDS-PAGE sample buffer and detected by immunoblot with anti-GST (p40PX) or anti-GFP (*P. falciparum* proteins). 10% of unbound fraction volume was loaded to visualize protein inputs. No binding was detected for *P. falciparum* exported proteins. Experiments were performed in triplicate.

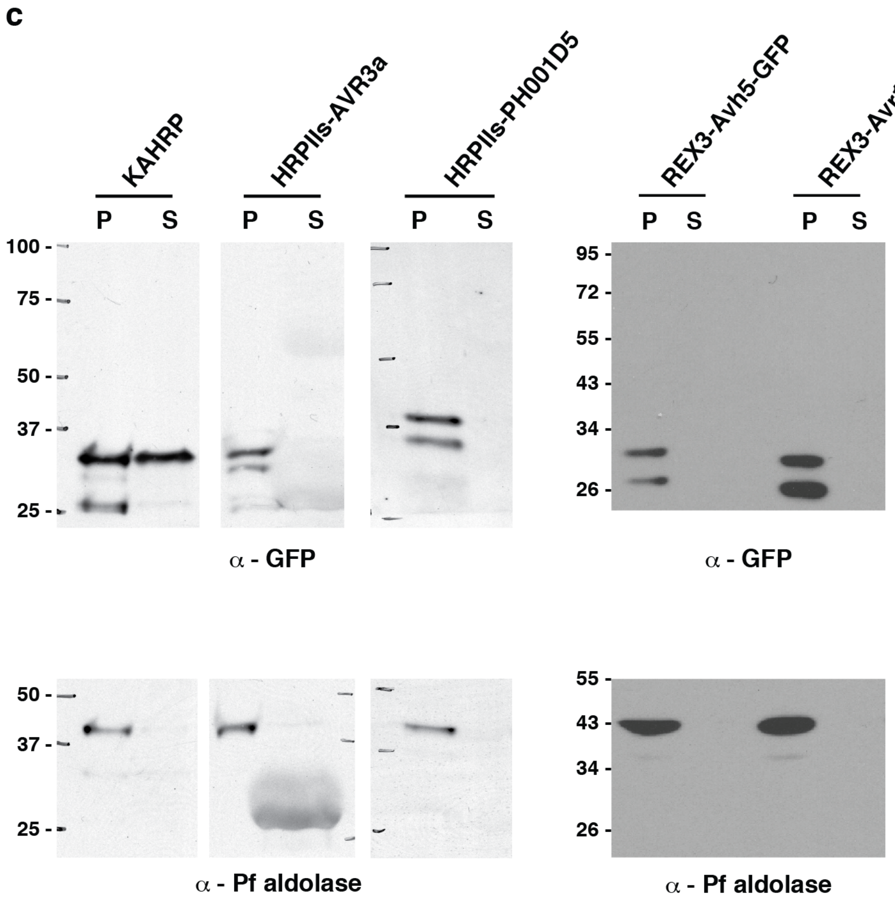

Supplementary Figure 8. Immunoblot with anti-GFP antibodies of each chimera from the tetanolysin pellet (P) and supernatant (S) shows that $KAHRP_{1-96}$ is exported but RxLR chimeras are not. Full-length blots from Fig. 3c. Tetanolysin inserts pores in the erythrocyte membrane allowing sampling of the host cell cytosol. Aldolase was included as a control, as described previously.

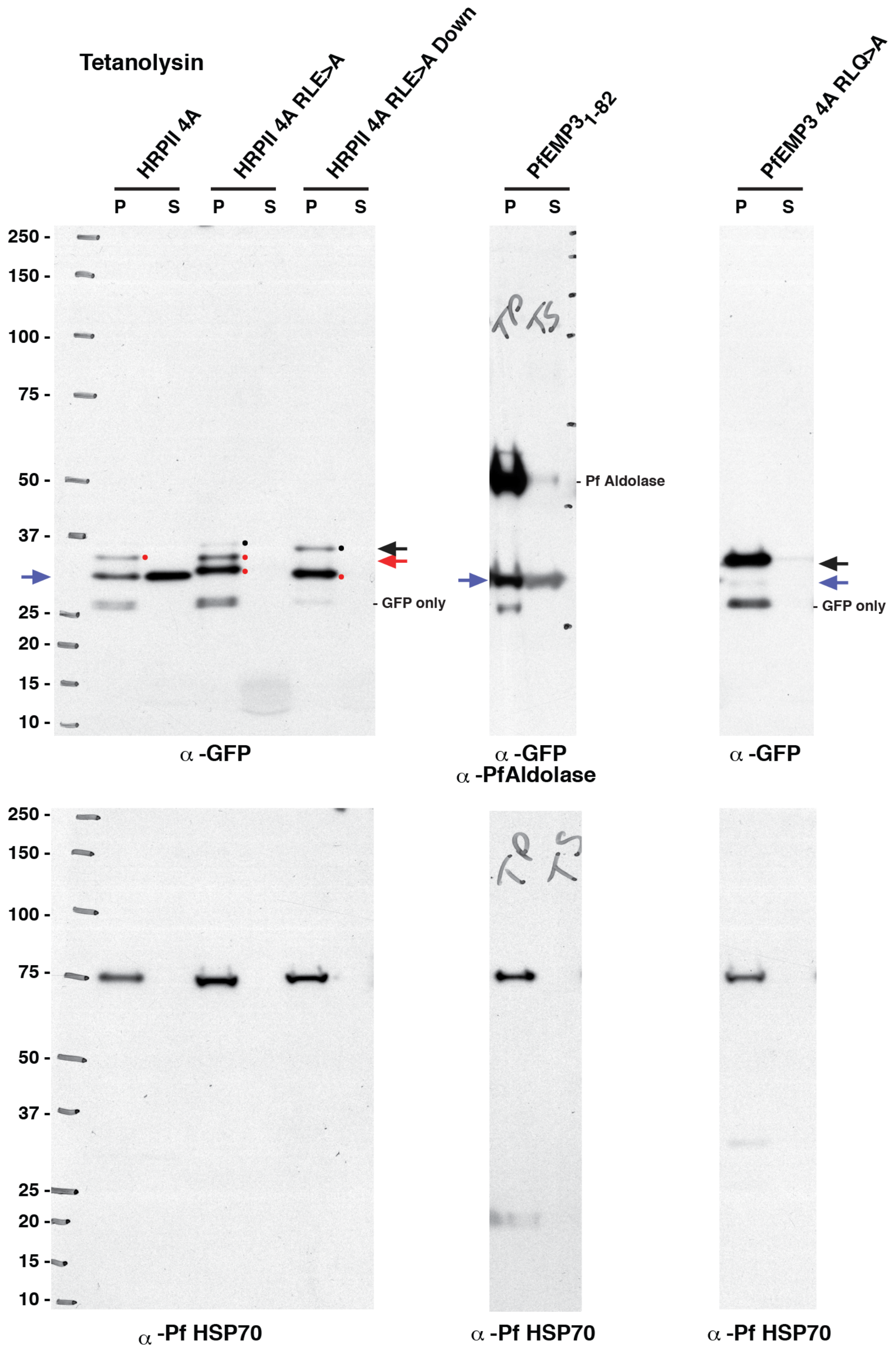

Supplementary Figure 9. Immunoblot with anti-GFP antibodies of chimeras from the tetanolysin pellet (P) and supernatant (S) shows that HRPII 4A and PfEMP3$_{1-82}$ are cleaved at the PEXEL (blue arrow) and exported. Full-length blots from Fig. 4d. Signal peptide-cleaved HRPII 4A was also visible (red spot). HRPII 4A RLE>A, HRPII 4A RLE>A Down, and PfEMP3$_{1-82}$ RLQ>A were not exported and bands corresponding to signal peptide-cleaved (various positions possibly due to insertion of four alanines; red spots/arrow) and uncleaved protein (black spots/arrow) are present.

c

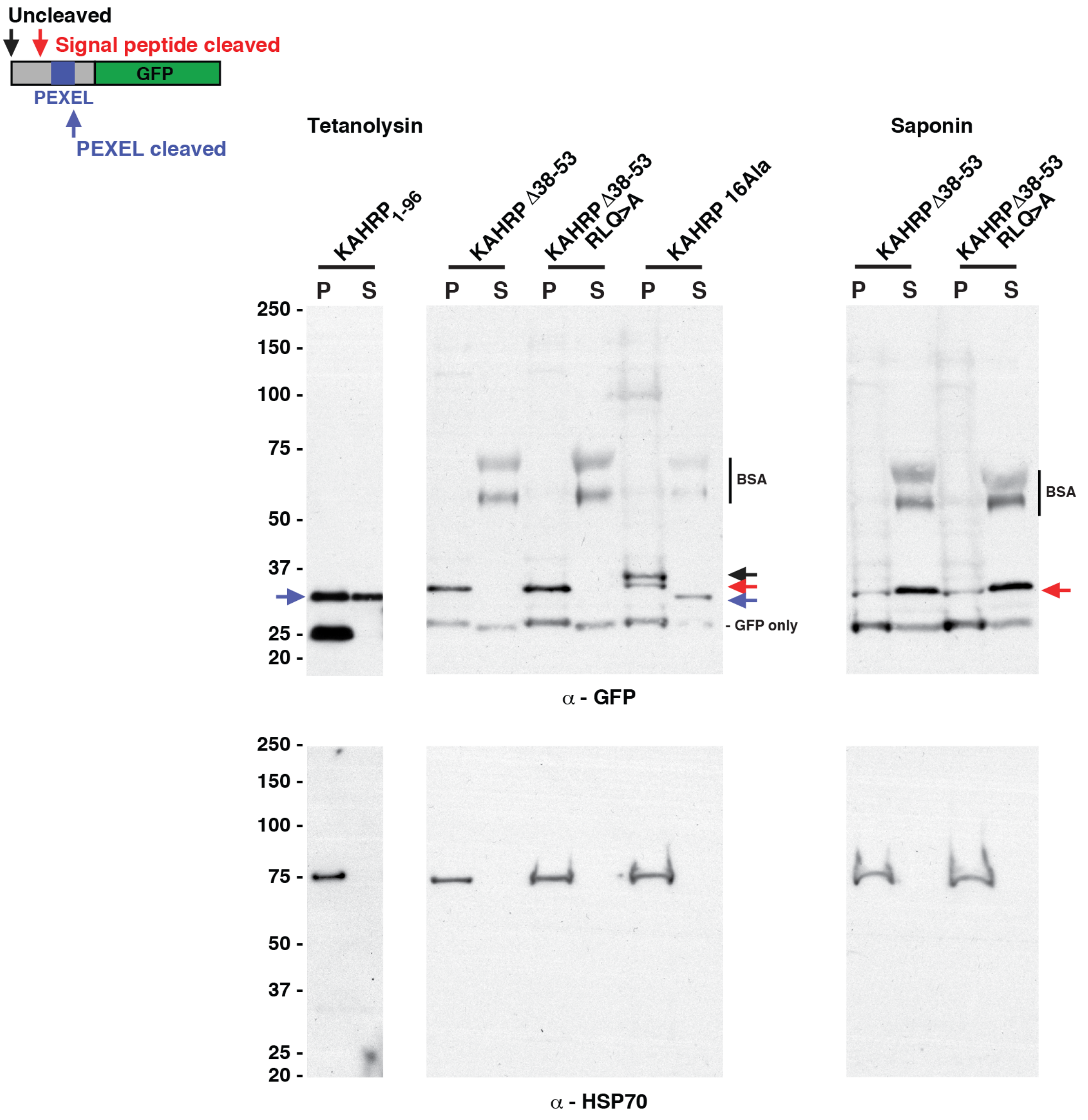

Supplementary Figure 10. Immunoblot with anti-GFP antibodies of chimeras from the tetanolysin and saponin pellet (P) and supernatant (S). Full-length blots from Fig. 5c. This shows KAHRP$_{1-96}$ at a size corresponding to cleavage at the PEXEL and it was exported (32 kDa; blue arrows), KAHRPΔ38-53 and KAHRPΔ38-53 RLQ>E were cleaved to a size corresponding to cleavage by signal peptidase (32.5 kDa; red spots) and were not exported, but were present in the saponin supernatant confirming they were secreted to the PV, and that KAHRP16Ala was of a size corresponding to cleavage at the PEXEL and it was exported (32 kDa; blue arrow) but cleavage was inefficient as bands consistent with signal sequence cleaved (red arrow) and uncleaved (black) species were present in the pellet fraction. HSP70 was used as a control.

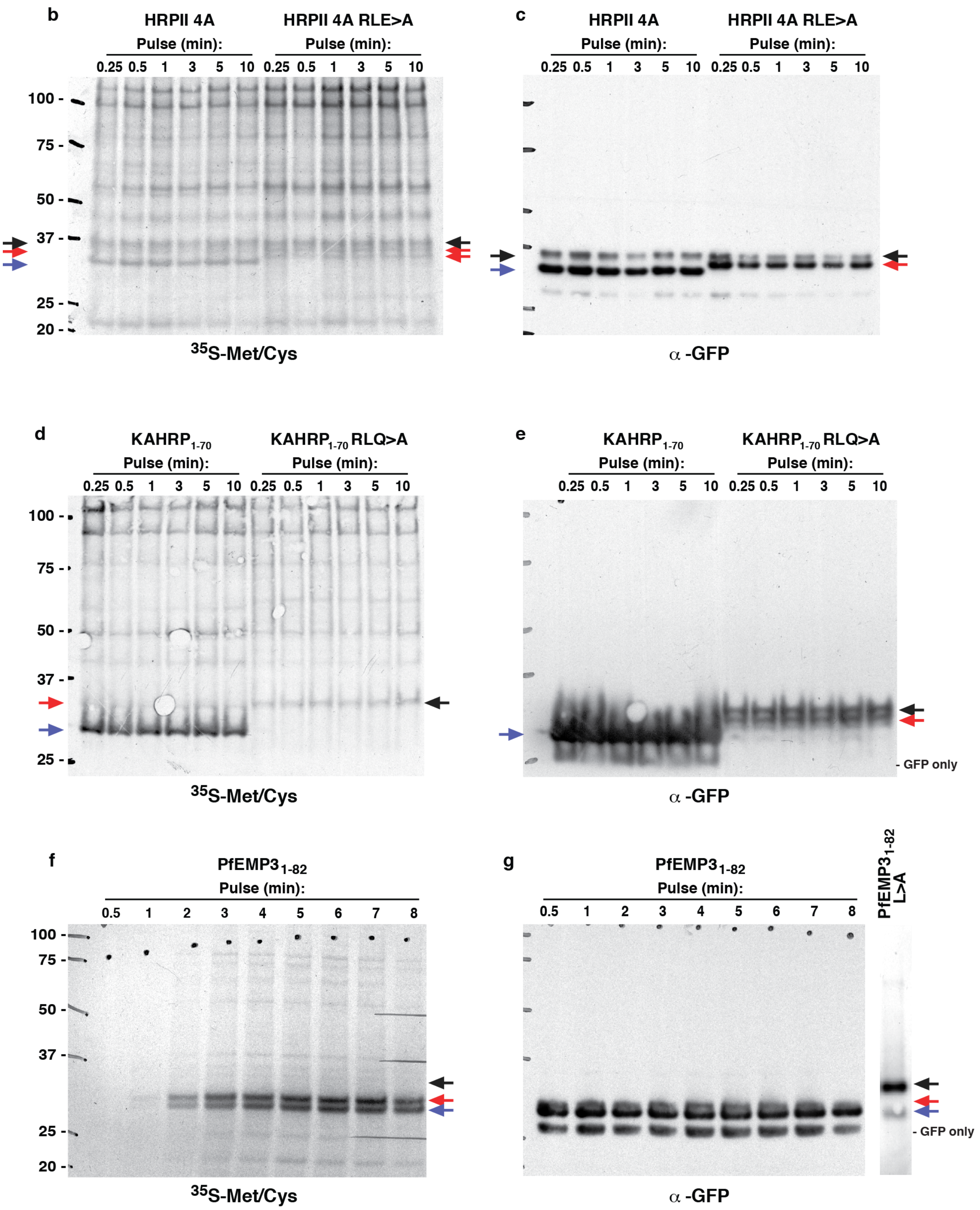

Supplementary Figure 11. The PEXEL is cleaved co-translationally. Full-length blots for Fig. 6b-g. **(b)** ^{35}S-Methionine/Cysteine-labelling (^{35}S-Met/Cys) of HRPII 4A and HRPII 4A RLE>A in *P. falciparum* for the indicated times shows bands corresponding to uncleaved (black arrow), signal peptide-cleaved (red arrow) and PEXEL-cleaved (blue arrow) species. Two signal peptide-cleaved species are present for RLE>A (red arrows), possibly due to insertion of four alanines following the signal sequence. **(c)** Immunoblot with anti-GFP

antibodies of the same membrane shows the bands are GFP-specific. The uncleaved HRPII 4A protein likely represents small quantities not detected by immunoblot as this reveals protein produced over hours. **(d)** ^{35}S-Met/Cys-labelling of $KAHRP_{1-70}$ and $KAHRP_{1-70}$-RLQ>A in *P. falciparum*. Bands corresponding to uncleaved (black arrow), signal peptide-cleaved (red arrow) and PEXEL-cleaved (blue arrow) species are indicated. **(e)** Immunoblot with anti-GFP antibodies of the same membrane. Note the absence of a signal peptidase cleaved product for KAHRP1-70 RLQ>A in the autoradiography. **(f)** Left: ^{35}S-Met/Cys-labelling of $PfEMP3_{1-82}$ in *P. falciparum*. Bands corresponding to cleavage of the indented signal peptide (red arrow) and PEXEL (blue arrow) are indicated. **(g)** Immunoblot with anti-GFP antibodies of the same membrane. A PEXEL mutant ($PfEMP3_{1-82}$ L>A) was included as a size control. Most protein detected with anti-GFP was present prior to radiolabelling so quantity is not comparable to ^{35}S-Met/Cys. Incorporation of ^{35}S-Met/Cys into HRPII and KAHRP constructs did not increase appreciably over time, in contrast to PfEMP3. This reason for this is unknown but may be due to saturation of anti-GFP agarose in the former experiments, which was used to enrich the GFP proteins.

Supplementary Tables

Supplementary Table 1: Summary of constructs tested in previous studies and this study

Previous construct	Previous result	Our construct	Our result	Same amino acid sequences ?
Avr3aGFP*	Exported	HRPIIss-AVR3a	Not exported	Yes
PH001D5GFP*	Exported	HRPIIss-PH001D5	Not exported	Yes
p40-phox-PX	Binds PI(3)P	P40PX	Binds PI(3)P	Yes
HRPII HT	Binds PI(3)P	HRPII WT	Low/no PI(3)P binding	Ours used 3D7 RLLHE PEXEL, previous used FCB RLLYE #
HRPII ALAYA	Low/no PI(3)P binding	HRPII RLE>A	Low/no PI(3)P binding	Ours used 3D7 ALAHA PEXEL, previous used FCB ALAYA #
PfEMP1 (PFL1960w) KELLD- GFP	Binds PI(3)P	PfEMP1 WT	Low/no PI(3)P binding	Yes
PfEMP1 (PFL1960w) AELLD-GFP	Low/no PI(3)P binding	PfEMP1 K>A	Low/no PI(3)P binding	Yes
SS-Nuk10WT-GFP	Exported	HRPIIss-PH001D5	Not exported	Similar but Nuk10 has 4Ala just after signal sequence
SS-AAAA-HT-GFP	Exported	HRPII 4A	Exported	Ours used 3D7 RLLHE PEXEL, previous used RLLYE #
SS-AAAA-ALAYA-GFP	Exported	HRPII 4A RLE>A	Not exported	Ours used 3D7 ALAHA PEXEL, previous used ALAYA #
SS-AAAA-ALAYA-down-GFP	Not exported	HRPII 4A RLE>A Down	Not exported	Ours used 3D7 ALAHA PEXEL, previous used ALAYA #
PfEMP3-RSLAQ-GFP	Exported	PfEMP3$_{1\text{-}82}$	Exported	Yes
PfEMP3-A4-ASAAA-GFP	Exported	PfEMP3 4A RLQ>A	Not exported	Yes
SS-p40phox^{WT}-mCherry	Retained in ER reportedly via	STEVORss-p40PX-GFP and	Both secreted to PV, not	Same p40 domain sequence, different signal

	PI(3)P binding	ACPss-p40PX -GFP	retained in ER	sequence and fluorescent tag
SS-p40phox^{R58Q}-mCherry	Secreted to PV, no longer ER	ACPss-p40PX$_{R58Q}$-GFP	Secreted to PV, not in ER	Same p40 domain sequence, different signal sequence and fluorescent tag

*These constructs are from Bhattacharjee *et al* PloS Pathogens 2006 and the remainder are from Bhattacharjee *et al* Cell 2012.
#The unusual PEXEL sequence RLLYE was used in Bhattacharjee *et al* Cell 2012 instead of the 3D7 PEXEL sequence RLLHE from *P. falciparum* 3D7 HRPII.
All studies used *P. falciparum* strain 3D7 for transfection and export analyses.

Supplementary Table 2: Oligonucelotide sequences used for making transfection constructs

Name	Oligonucleotide sequence (underlined bases are restriction sites for cloning)
JB170	GAGCATATGTACATCTGCGGTGAC
JB171	ATACCGCGGCAGGTCTTCTTCAGAGATCAGTTTCTGTTCA GATTCACGGAAACCGT AGTCA
JB172	GAGCATATGCGTATCCTGGCTGAAGGTGA
JB173	TGAACCCCTCCGCGGCAGGT
JB174	ATACCGCGGCAGGTCTTCTTCAGAGATCAGTTTCTGTTCT TCAGCCAGGATACGAGATT
JB175	GAGCATATGGGTGAAGACACCTGCGCTCG
JB176	AGACCGCGGAGTAAAGGAGAAGAACTTTTCA
JB177	AGAGGATCCATGATGATGATGGTGGTGATGATGTTTGTA TAGTTCATCCATGCCA
P40_A_F	GGCCTAGGATGGCTGTGGCCCAGCAG
p40_K_R	CCGGTACCGCGGAGTGCCTGGGGCAC
GFP_K_F	GTACGGTACCATGAGTAAAGGAGAAGAAC
GFP_SDEL_X_R	GGCCCGGGTTAAAGTTCATCACTTTTGTATAGTTCATCCA TGCCA
5'ACPsp-PX40p	TATATATAATGAAGATCTTATTACTTTGTATAATTTTTCT ATATTATGTTAACGCTTTTAAAAATACAATGGCTGTGGCC CAGCAGCTG
3'KpnI-PX40-rev	CCCGGTACCTGAGTCATAGGGCGACTGG
HRS_FYVE_AvrII_F	GGCCTAGGATGGAAAGTGATGCCATGTTCG
HRS_FYVE_KpnI_R	CCGGTACCTGCCTTCTTGTTCAGCTGCTC
Rex3(1-43)-Avr1b(22-71)KpnI-rev	CAGTCCTAGGATCTGTTACTGAAAATGTTCTTTCACCAGC ATCATCTTCTTCATGAGCTCTTAAAAATCTACCACCAGCT ATATCACCATTTCTTAATGATCTTCTTACTAAATCTGGTG ATTCTACCATAGCTATATTTGTTTCATCTGAATATTCTGT GCTCCCTTCGTATGAGGTGGTATATTTATAG
Rex3Avh5(20-63)-avrII-rev	CAGTCCTAGGTTTACCTGGATTATGTACTTTTGGTTCATA TACTATATCTGTATCAGCTGTTCTTAAAAATCTTCTTGAT CTTGTTACTGTTTGTACTGGAGCATTTACTGATTGTAAAT TAGCATCATCTGGTACTCTTGTGCTCCCTTCGTATGAGGT GGTATATTTATAG

Supplementary References

1. Bhattacharjee S, Stahelin RV, Speicher KD, Speicher DW, Haldar K. Endoplasmic reticulum PI(3)P lipid binding targets malaria proteins to the host cell. *Cell* **148**, 201-212 (2012).

2. Boddey JA, *et al.* An aspartyl protease directs malaria effector proteins to the host cell. *Nature* **463**, 627-631 (2010).
